# The Multifaceted Role of Pyroptosis: Molecular Mechanisms, Crosstalk with Other Cell Death Pathways, and Therapeutic Implications

**DOI:** 10.1002/mco2.70844

**Published:** 2026-07-07

**Authors:** Diego Liviu Boaru, Patricia De Castro‐Martinez, Cielo Garcia‐Montero, Oscar Fraile‐Martinez, Majd N. Michael Alhaddadin, Silvestra Barrena‐Blázquez, Laura Lopez‐Gonzalez, Clara Tasende Fernández, Ricardo Alvarado‐Hurtado, Tomás Ratia Giménez, Inmaculada Lasa‐Unzúe, Manuel Díez Alonso, Alberto Gutiérrez‐Calvo, Miguel A. Saez, David Cobo‐Prieto, Jose V. Saz, Melchor Alvarez‐Mon, Raul Diaz‐Pedrero, Miguel A. Ortega

**Affiliations:** ^1^ Department of Medicine and Medical Specialties Faculty of Medicine and Health Sciences Network Biomedical Research Center for Liver and Digestive Diseases (CIBEREHD) University of Alcalá Alcalá de Henares Spain; ^2^ Ramón y Cajal Institute of Sanitary Research (IRYCIS) Madrid Spain; ^3^ Department of Nursing and Physiotherapy Faculty of Medicine and Health Sciences University of Alcalá Alcalá de Henares Spain; ^4^ Department of Surgery Medical and Social Sciences Faculty of Medicine and Health Sciences University of Alcalá Alcalá de Henares Spain; ^5^ Endocrinology and Nutrition Service Hospital Universitario Príncipe De Asturias (CIBEREHD) Alcalá de Henares Spain; ^6^ Department of General and Digestive Surgery Príncipe De Asturias University Hospital Alcala de Henares Spain; ^7^ Pathological Anatomy Service Central University Hospital of Defense‐UAH Madrid Alcala de Henares Spain; ^8^ Immune System Diseases‐Rheumatology Oncology Service and Internal Medicine (CIBEREHD) University Hospital Príncipe De Asturias Alcala de Henares Spain

**Keywords:** apoptosis, cancer, extracellular vesicles, nanoparticles, PANoptosis, pyroptosis

## Abstract

Pyroptosis is a tightly regulated, proinflammatory form of programmed cell death defined by gasdermin‐mediated membrane pore formation and cytokine release. By coupling cell death to innate immune activation, pyroptosis occupies a unique position at the interface of host defense and tissue pathology. Recent advances have demonstrated that pyroptosis is activated through multiple molecular routes, including canonical and noncanonical inflammasome pathways, Caspase‐3/8–gasdermin axes, and granzyme‐dependent mechanisms. Importantly, pyroptosis does not operate in isolation but engages in extensive crosstalk with other programmed cell death modalities, such as apoptosis, necroptosis, ferroptosis, NETosis, and the integrative process of PANoptosis, sharing key regulators including caspases, inflammasomes, and gasdermins. Despite rapid progress, a unified framework integrating pyroptosis‐specific mechanisms with interaction networks, temporal dynamics, and disease relevance is still lacking. In this review, we systematically dissect the molecular pathways governing pyroptosis, emphasize its functional interplay with other cell death programs, and discuss emerging concepts such as cell‐type specificity and sublytic versus lytic outcomes. We further examine the contribution of pyroptosis to cancer, infectious, autoimmune, metabolic, and cardiovascular diseases, and critically evaluate therapeutic strategies aimed at either inhibiting excessive pyroptosis or exploiting it for immunogenic cell death. Collectively, this review offers a perspective‐enhancing, mechanistic, and translational significance.

## Graphical abstract

Pyroptosis maintains immune homeostasis by eliminating damaged or infected cells, but its dysregulation promotes inflammation and cancer progression. The diagram illustrates key activation pathways, links with other programmed cell deaths, and cancer‐specific effects, enhancing its dual protective and pathogenic roles.

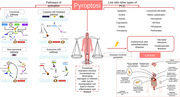

## 1 Introduction

Programmed cell death (PCD) is a tightly orchestrated biological process governed by genetically encoded pathways that ensure proper development, tissue homeostasis, and immune regulation [1]. Since its initial description in the 1960s [2], the concept of PCD has expanded far beyond apoptosis to encompass a diverse spectrum of regulated cell death modalities, including ferroptosis, pyroptosis, and, more recently, described mechanisms such as cuproptosis [3, 4]. These pathways are characterized by distinct molecular signatures and morphological features, yet growing evidence indicates that they operate as interconnected and highly adaptable signaling networks rather than isolated programs [5]. Dysregulation of PCD is now recognized as a central driver of numerous pathological conditions, including cancer, autoimmune disorders, neurodegeneration, metabolic disease, and infection [6, 7]. These processes are orchestrated by intricate intracellular and extracellular cues that converge on molecular cascades to determine cell fate [8].

Within this expanding landscape, pyroptosis has emerged as a particularly distinctive and clinically relevant form of regulated cell death [9]. Unlike apoptosis, which is generally immunologically silent, pyroptosis is intrinsically proinflammatory and is characterized by gasdermin‐mediated membrane pore formation, cellular swelling, and the release of cytokines such as interleukin (IL)‐1β and IL‐18 [10, 11].

Despite the rapid growth of the field, current literature often addresses pyroptosis in a fragmented manner, focusing either on its molecular machinery or on disease‐specific contexts. In particular, a comprehensive framework integrating pyroptosis with other PCD modalities, such as apoptosis, necroptosis, ferroptosis, NETosis, and the integrative process of PANoptosis, remains limited [12, 13]. Moreover, emerging concepts, including cell type specificity, temporal regulation, and the distinction between sublytic and lytic pyroptotic outcomes, have not yet been systematically synthesized. Addressing these gaps is necessary to fully understand the biological significance of pyroptosis and its role within the broader regulated cell death network.

**FIGURE 1:**
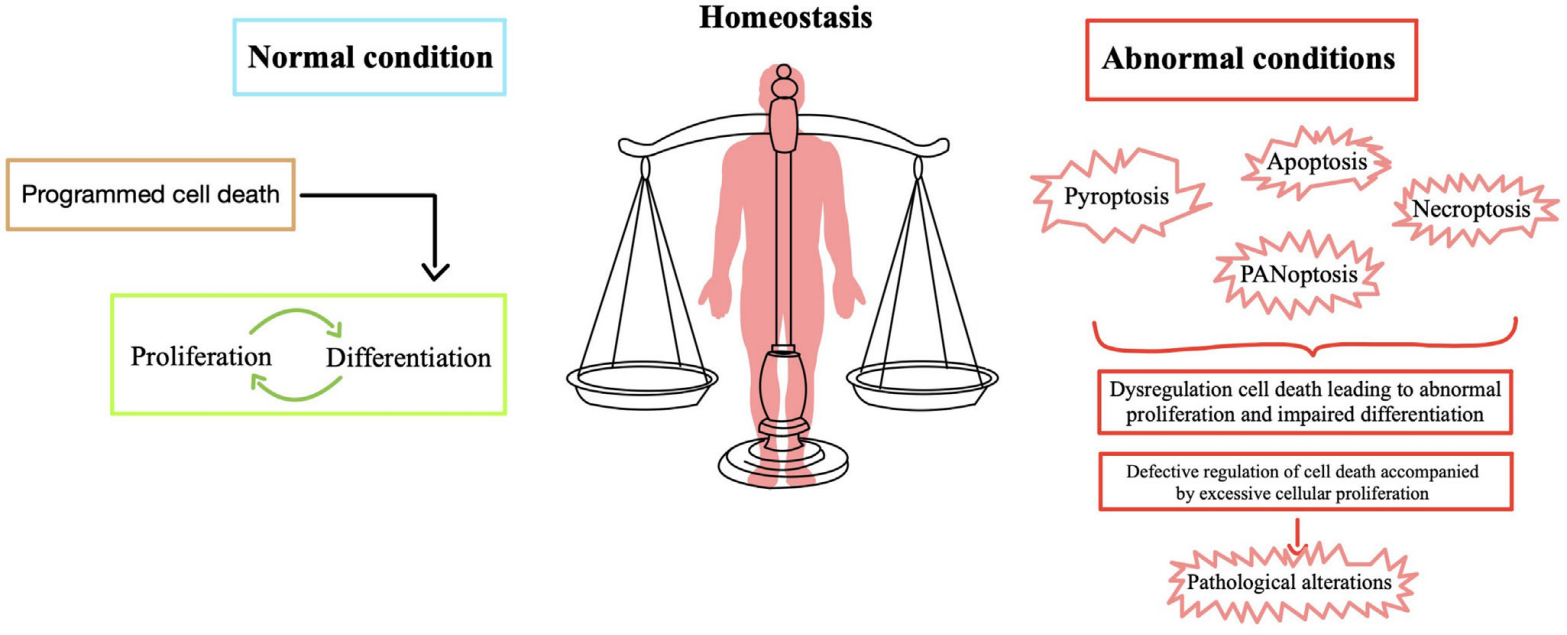
Homeostatic balance of programmed cell death pathways in human health and disease. Under physiological conditions, regulated programmed cell death (including pyroptosis, apoptosis, necroptosis, and PANoptosis) preserves tissue homeostasis by coordinating cell turnover and immune surveillance. Dysregulation or excessive activation of these pathways disrupts this balance, leading to aberrant cell death, impaired differentiation, chronic inflammation, and disease development, including cancer. Pyroptosis exemplifies how inflammatory cell death can be protective or pathological depending on its molecular control and context.

In this review, we first provide an overview of the main forms of PCD to establish a conceptual framework for understanding pyroptosis in context. Then, the molecular mechanisms underlying canonical and noncanonical pyroptosis pathways are described in detail, including upstream regulatory signals and gasdermin‐mediated execution. Next, the functional crosstalk between pyroptosis and other regulated cell death programs is examined, enhancing shared molecular regulators and convergence points such as PANoptosis. Finally, the role of pyroptosis across cancer, infectious, autoimmune, metabolic, and cardiovascular diseases is analyzed, and current and emerging therapeutic strategies aimed at modulating pyroptosis are discussed, underscoring its translational relevance and future research potential (Figure 1).

## 2 Different Types of PCD

PCD encompasses a diverse set of genetically regulated mechanisms that differ in molecular execution, morphological features, and biological consequences. Although historically studied as independent processes, it is now evident that multiple forms of PCD, including apoptosis, necroptosis, ferroptosis, autophagy‐dependent cell death, and pyroptosis, are interconnected through shared signaling nodes and regulatory checkpoints. A systemic overview of these pathways is essential to contextualize pyroptosis within the broader regulated cell death network, and to understand how distinct death programs cooperate, compensate, or antagonize one another under physiological and pathological conditions.

### 2.1 Apoptosis

Apoptosis, often described as a cellular “suicide program,” is a tightly regulated form of PCD essential for maintaining tissue integrity [14]. It is mediated by the caspase family of proteases, which operate through two main pathways: the intrinsic (mitochondrial) route and the extrinsic (death receptor‐mediated) route [15, 16]. Morphologically, apoptotic cells exhibit nuclear condensation, chromatin fragmentation, and the formation of apoptotic bodies, which are subsequently cleared without triggering inflammation [17]. In the intrinsic pathway, mitochondrial outer membrane permeabilization leads to the release of cytochrome *c* and the assembly of the apoptosome, activating Caspase‐9 and, in turn, the executioner Caspases‐3 and ‐7 [18]. The extrinsic pathway, by contrast, is initiated when death receptors such as Fas or TNFR engage their ligands, forming the DISC complex and activating Caspase‐8 [19]. Both pathways converge on executioner caspases, which dismantle the cell in a controlled manner [20]. Importantly, apoptosis is immunologically silent compared with pyroptosis or necroptosis, enhancing its role in removing damaged or potentially harmful cells without provoking inflammation [21]. Dysregulation of this process contributes to cancer, autoimmune disease, and neurodegeneration, underscoring its significance as both a physiological safeguard and a therapeutic target.

Beyond its classical role in tissue homeostasis, apoptosis is increasingly recognized as an immunologically nuanced process [22]. While apoptotic cells are typically cleared in an anti‐inflammatory manner through efferocytosis, under certain contexts, apoptosis can become immunogenic, particularly when accompanied by endoplasmic reticulum stress, mitochondrial dysfunction, or defective corpse clearance, contributing to damage‐associated molecular pattern (DAMP) exposure (e.g., calreticulin) and downstream immune activation [23].

### 2.2 Anoikis

Anoikis, first described by Frisch in 1994, is a specialized form of apoptosis triggered when cells lose their attachment to the extracellular matrix (ECM) [24]. This “homelessness” response acts as a protective mechanism, preventing displaced cells from surviving or proliferating in unsuitable environments, thereby safeguarding tissues against dysplastic growth [25]. The process is mediated by integrins, which normally transmit survival signals through cytoskeletal and signaling pathways [26, 27]. When these signals are lost, both the intrinsic (mitochondrial‐mediated) and extrinsic (death receptor‐mediated) apoptotic pathways converge, leading to caspase activation and cell death [27, 28]. Central regulators include members of the Bcl‐2 family, with proapoptotic and antiapoptotic proteins finely balancing survival and death signals [29]. Importantly, defective anoikis allows detached cells to resist apoptosis, a hallmark of cancer progression that facilitates invasion and metastasis [30]. Thus, anoikis not only exemplifies the link between adhesion and survival but also represents a key barrier whose evasion underlies tumor dissemination.

Mechanistically, anoikis is governed by integrin‐dependent survival signaling, prominently via FAK–SRC and PI3K/AKT pathways [31], while transcriptional programs such as Hippo–YAP/TAZ can rewire mechanotransduction to sustain survival in detached cells [32]. These adaptations underpin anoikis resistance during metastasis and are now viewed as both biochemical and biophysical processes shaped by matrix stiffened and cytoskeletal tension [33].

### 2.3 Autosis (Autophagy‐Dependent Cell Death)

Autosis is a recently identified form of PCD that arises from excessive or dysregulated autophagy and uniquely depends on Na^+^/K^+^‐ATPase activity [34]. Unlike apoptosis or necrosis, it presents with distinct morphological changes, including nuclear membrane convolution, endoplasmic reticulum dilation, mitochondrial alterations, and perinuclear ballooning, alongside increased adhesion of dying cells to their substrate [35, 36]. Autosis has been observed in multiple pathological settings, such as cerebral and cardiac ischemia/reperfusion, renal injury, viral infections, and skin differentiation, suggesting a wide physiological relevance [34, 37]. Remarkably, it shows cell‐type selectivity, preferentially affecting stressed or diseased cells such as HIV‐infected T cells and tumor cells, while sparing normal tissues [38]. This selective vulnerability is linked to altered autophagy machinery and variations in Na^+^/K^+^‐ATPase regulation across cell types [39]. Pharmacological modulation of autosis, including induction with Tat–BECN1 peptide or inhibition by cardiac glycosides, has revealed its potential as a therapeutic strategy [40, 41]. Harnessing autosis to eliminate infected or malignant cells without harming healthy ones opens a promising frontier in both infectious disease and cancer therapy.

Autosis has been linked to ischemia/reperfusion injury and neuronal stress, where excessive autophagy flux and Na^+^/K^+^‐ATPase regulation occur across cell types [34, 42]. Pharmacological modulation of autosis, including induction with Tat–BECN1 peptide or distinctive death morphology (perinuclear space ballooning) [43]. Importantly, pharmacological inhibition with cardiac glycosides enhances a potentially druggable checkpoint, supporting the idea that autophagy can be prodeath in specific cellular states rather than uniformly cytoprotective [44, 45].

### 2.4 Cuproptosis

Cuproptosis is a recently described form of regulated cell death that depends on intracellular copper accumulation [46]. Unlike ferroptosis, this pathway is initiated when copper ions are transported into the mitochondria and enzymatically reduced by FDX1 [47]. The reduced copper binds to lipoylated components of the tricarboxylic acid cycle, such as DLAT, leading to their aggregation and destabilization of iron–sulfur cluster proteins [48]. This process triggers intense proteotoxic stress rather than oxidative damage, distinguishing cuproptosis from other metal‐driven deaths [49]. Cells with high reliance on oxidative phosphorylation are particularly vulnerable, while glycolytic cells show resistance [50]. Pharmacological studies using copper ionophores like elesclomol have demonstrated potent anticancer effects, which can be reversed by copper chelators but not by inhibitors of other death pathways [51]. This unique mitochondrial vulnerability positions cuproptosis as both a promising therapeutic target in oncology and a novel frontier in the study of metal biology.

Cuproptosis is tightly coupled to mitochondrial respiration and protein lipoylation. Copper binding to lipoylated TCA‐cycle enzymes promotes toxic aggregation and loss of Fe–S cluster proteins, triggering proteotoxic stress rather than classical ROS‐driven injury [52]. This metabolic selectivity helps explain why oxidative phosphorylation‐dependent tumors may be particularly vulnerable to copper ionophores, positioning cuproptosis as an emerging anticancer strategy.

### 2.5 Entosis

Entosis represents a unique form of regulated cell death characterized by live cell engulfment [53]. Unlike apoptosis, which relies on self‐destruction, entotic cells are killed nonautonomously after being internalized by neighboring host cells, creating striking cell‐in‐cell structures [54]. This process is driven by actin–myosin contractility regulated by RHOA–ROCK signaling and stabilized by cadherin‐based junctions, which direct invasive forces that push one cell into another [55]. Once internalized, most endocytic cells are ultimately degraded through lysosome‐dependent mechanisms, although some may temporarily survive or even escape [54]. Importantly, entosis has been closely associated with cancer, where it can fuel competition between cells, provide nutrients to host cells under metabolic stress, and promote genomic instability through cytokinesis failure [56]. Thus, while entosis eliminates certain cells, its broader impact may paradoxically favor tumor progression, positioning it as a complex and context‐dependent form of PCD.

Entosis is often triggered by matrix detachment, glucose starvation, or mitotic stress and is executed through RHOA–ROCK–actomyosin contractility, producing cell‐in‐cell structures [57]. In tumors, entosis can mediate cell competition when host‐cell cytokinesis fails, emphasizing its context‐dependent role in cancer evolution [58].

### 2.6 Ferroptosis

Ferroptosis is a distinct and tightly regulated form of cell death that differs from apoptosis and other classical pathways by its reliance on iron and lipid peroxidation [59]. Unlike caspase‐dependent mechanisms, ferroptosis is driven by an accumulation of reactive oxygen species (ROS) and uncontrolled oxidative damage to membrane lipids, ultimately leading to loss of cellular integrity [60]. Morphologically, it is characterized by shrunken mitochondria, increased membrane density, and organelle swelling. Functionally, ferroptosis plays dual roles: under physiological conditions, it contributes to tissue homeostasis, but when dysregulated, it is strongly implicated in cancer, neurodegeneration, and ischemia–reperfusion injury [61, 62, 63]. Its initiation is closely linked to redox imbalance, iron metabolism, and the failure of antioxidant defenses such as glutathione peroxidase 4 (GPX4) [64]. Recent research highlights that ferroptosis can act both as a tumor‐suppressive mechanism and as a driver of disease progression, positioning it as a promising yet complex therapeutic target [65].

Ferroptosis is orchestrated by the failure of lipid peroxide detoxification, classically through impairment of the system Xc‐/GSH/GPX4 axis, and is amplified by iron‐dependent radical chemistry and PUFA‐phospholipid oxidation [66]. Additional protective modules (e.g., FSP1–CoQ10 and GCH1–BH4 pathways) further illustrate that ferroptosis sensitivity is shaped by parallel antioxidant networks, with string implications for therapy resistance and ischemia–reperfusion damage [67].

### 2.7 Lysosomal Cell Death

Lysosomal cell death (LCD) represents a unique and evolutionarily conserved form of regulated cell death, in which lysosomal membrane permeabilization (LMP) releases hydrolytic enzymes, particularly cathepsins, into the cytosol, triggering downstream apoptotic or necrotic pathways [68]. Originally described by de Duve as suicide bags, lysosomes can mediate cell death when destabilized by various stimuli, including ROS, lysosomotropic agents, and proapoptotic Bcl‐2 proteins [69]. The extent of LMP dictates the outcome: extensive rupture induces necrosis, whereas limited permeabilization can initiate apoptosis‐like processes [70]. Lysosomal enlargement, membrane composition, and chaperones such as Hsp70 modulate LMP susceptibility, highlighting a complex regulatory network [71]. LCD also intersects with other death modalities, including ferroptosis, pyroptosis, autophagy‐dependent death, and entosis [72]. Therapeutically targeting lysosomal stability, either to enhance LMP in cancer cells or to stabilize lysosomes in degenerative diseases, offers promising strategies, as exemplified by lysomotropic and cationic amphiphilic drugs [73, 74].

Lysosome‐dependent death is governed by the degree and kinetics of LMP, which can range from limited, spatially restricted cathepsin leakage (compatible with survival signaling) to catastrophic rupture driving apoptosis‐like or necrotic outcomes [75, 76]. This graded behavior links lysosomal integrity to inflammation and to inflammasome priming in sterile injury and infection [77].

### 2.8 Mitotic Catastrophe

Mitotic catastrophe is a regulated oncosuppressive process that serves as a safeguard against the propagation of genetically unstable cells [78]. Initially observed in the early 20th century, it was later defined as a cellular response to mitotic failure caused by DNA damage, spindle dysfunction, or checkpoint impairment [79, 80]. Rather than being an independent form of PCD, mitotic catastrophe represents a prelude to various lethal outcomes such as apoptosis, necrosis, or autophagy [81]. Its hallmark features include prolonged mitotic arrest, chromosome missegregation, and the formation of multinucleated or micronucleated cells due to failed cytokinesis [82]. Molecularly, this process is regulated by p53, SAC components, and mitotic kinases such as Aurora B and Wee1, whose dysfunction enhances chromosomal instability [83]. Therapeutically, inducing mitotic catastrophe has emerged as a strategy to eliminate tumor cells resistant to conventional apoptosis, although some cells may survive, undergo senescence, or escape via entosis, contributing to tumor resistance [84].

Mitotic catastrophe is now widely viewed as an oncosuppressive stress response rather than a single execution program: prolonged spindle checkpoint activation, centrosome amplification, or DNA damage can culminate in apoptosis, necroptosis, senescence, or even entosis, depending on p53 status and checkpoint wiring [85]. This plasticity is therapeutically relevant because many antimitotic agents eliminate tumor cells through postmitotic death rather than immediate apoptosis [86].

### 2.9 Necroptosis

Necroptosis represents a distinct form of PCD that bridges apoptosis and necrosis [87]. Unlike apoptosis, it proceeds independently of caspases and is orchestrated by the kinase cascade involving RIPK1, RIPK3, and MLKL [88]. Upon activation by death receptors such as TNFR1, TLRs, or interferon receptors, RIPK1 and RIPK3 assemble into the necrosome, leading to MLKL phosphorylation and its translocation to the plasma membrane, where membrane rupture occurs [89]. These processes release intracellular components that can trigger strong inflammatory and immune responses. Functionally, necroptosis serves as both a host defense mechanism against pathogens and a driver of tissue injury under pathological conditions [90]. In cancer, its role is context‐dependent, acting as a tumor suppressor through immunogenic cell death or promoting tumor progression via chronic inflammation [91]. Current therapeutic research is exploring selective inhibitors and inducers of necroptosis to modulate these dual outcomes, offering new opportunities for disease intervention [92].

Necroptosis is regulated by posttranslational modifications (phosphorylation and ubiquination) that tune RIPK1/RIPK3 complex assembly and MLKL activation [93]. Its inflammatory output also intersects with innate immune signaling, as necroptotic membrane rupture can amplify cytokine production and inflammasome activation, reinforcing the concept of regulated necrosis as an immune‐modulatory process [94].

### 2.10 NETosis

Neutrophils represent the most abundant subset of myeloid leukocytes, constituting nearly two‐thirds of circulating white blood cells [95]. Originating from hematopoietic stem cells in the bone marrow, they mature and enter the bloodstream as highly specialized phagocytes equipped for rapid immune defense [96]. Despite their short lifespan of roughly 3 days, neutrophils play a critical frontline role in host protection, swiftly migrating to sites of infection or tissue injury [97]. Upon activation, they unleash a broad antimicrobial arsenal, including granule enzymes, ROS, and the release of neutrophil extracellular traps (NETs), web‐like chromatin structures decorated with microbial proteins [98]. This unique process, known as NETosis, can proceed via suicidal or vital pathways depending on the stimulus and context [99]. Beyond infection, NETosis has emerged as a key player in inflammatory and autoimmune conditions, highlighting its dual nature as both a protective and potentially pathogenic mechanism within innate immunity [100].

NET formation can occur through lytic (“suicidal”) NETosis or through nonlytic (“vital”) pathways in which neutrophils remain functional. Key regulators include NADPH oxidase in mitochondrial ROS, PAD4‐mediated histone citrullination, and granular enzymes (NE, MPO). Dysregulated NETosis is increasingly implicated in immunothrombosis, sepsis, and autoimmunity, linking cell‐death‐like programs to vascular pathology.

### 2.11 PANoptosis

PANoptosis is a distinctive form of PCD that integrates apoptosis, pyroptosis, and necroptosis within a single, coordinated process [101]. This modality is orchestrated by the PANoptosome, a multiprotein complex that assembles important molecules from each pathway, including Caspase‐8, RIPK1/RIPK3, ASC, and gasdermins, to drive simultaneous cell death and inflammatory responses [102, 103]. Unlike classical cell death pathways, PANoptosis cannot be fully explained by apoptosis, pyroptosis, or necroptosis alone [104]. It is triggered by diverse stimuli, such as microbial infections, cytokine signaling, or cellular stress, leading to activation of multiple caspases, inflammasomes, and necroptotic effectors in parallel [105, 106]. This cross‐regulation ensures robust immune signaling, amplifying the release of cytokines and DAMPs. Emerging studies have linked PANoptosis to viral, bacterial, and fungal infections, as well as cancer, autoinflammatory, and neurodegenerative diseases [107, 108].

PANoptosis provides a unifying framework for inflammatory death programs in which pyroptotic, apoptotic, and necroptotic machineries are coactivated through PANoptosome assemblies (e.g., Caspase‐1 or MKLK), which may fail to prevent death in infection or cytokine storm settings, because the system reroutes execution across interconnected modules.

### 2.12 Parthanatos

Over recent decades, scientific advances have revealed a growing array of distinct death programs defined by specific molecular signatures and biochemical events. Among these, parthanatos has emerged as a unique caspase‐independent pathway driven by excessive activation of poly(ADP‐ribose) polyerase‐1 (PARP‐1) [109]. This hyperactivation leads to the overproduction of poly(ADP‐ribose) (PAR) polymers, mitochondrial depolarization, and nuclear translocation of apoptosis‐inducing factor (AIF), culminating in large‐scale DNA fragmentation and cell death [110]. Unlike apoptosis, parthanatos cannot be prevented by an‐caspase inhibitors and lacks apoptotic body formation, reflecting its distinct mechanistic nature [111]. Experimental evidence implicates parthanatos in numerous pathological contexts, including ischemia, neurodegeneration, diabetes, and cancer [112].

Parthanatos is driven by PARP‐1 hyperactivation and PAR polymer accumulation, promoting AIF release and large‐scale DNA fragmentation. Because this pathway is coupled to NAD^+^/ATP depletion and metabolic collapse, it is particularly relevant in ischemic and neurodegenerative settings, and it provides a rationale for neuroprotection via PARP inhibition in selected contexts.

### 2.13 Pyroptosis

Pyroptosis represents a highly regulated and proinflammatory form of PCD that serves as a crucial component of the innate immune responses. This process can be initiated through four major pathways: canonical, noncanonical, Caspase‐3/GSDME‐dependent, and granzyme‐mediated—each converging on the activation of gasdermin family members that form membrane pores [113]. In the canonical route, the inflammasome complexes such as NLRP3 and AIM2 activate Caspase‐1, promoting cleavage of gasdermin D (GSDMD) and maturation of IL‐1β and IL‐18. The noncanonical pathway, in contrast, relies on Caspase‐4/5/11, which directly senses cytosolic lipopolysaccharide (LPS) to trigger pyroptotic signaling [114, 115]. Beyond inflammasome control, apoptotic Caspase‐3 can also induce pyroptosis through GSDME activation, particularly in response to chemotherapeutic stress [116]. Moreover, immune effector cells, including cytotoxic T lymphocytes and NK cells, execute pyroptosis via granzymes A and B, which cleave GSDMB and GSDME, amplifying inflammatory responses within the tumor microenvironment [117]. Central to all these mechanisms are gasdermin, pore‐forming proteins that act as molecular switches between apoptosis and pyroptosis [118].

Collectively, these pathways reveal the intricate balance between host defense and inflammation, positioning pyroptosis as a key intersection between immune activation, tissue injury, and disease progression.

Table 1 compares all the PCDs based on their mechanistic way of action.

**TABLE 1:** Summary of major programmed cell death modalities.

PCD	Typical triggers	Key mediators	Morphology	Immune profile	Pathological relevance	References
Apoptosis	Intrinsic (MOMP) or extrinsic death	Caspase‐8/9 → Caspase‐3/7; BCL‐2 family	Cell shrinkage; apoptotic bodies	Generally noninflammatory; can be immunogenic in specific contexts	Cancer, autoimmunity, neurodegeneration	[15, 16]
Anoikis	ECM detachment	Integrins, FAK/SRC, PI3K/AKT; Caspase‐8/9/3	Apoptotic morphology in detached cells	Typically silent; resistance promotes metastasis	Metastasis, fibrosis	[27, 28]
Autosis	Excessive autophagy/Tat‐Beclin1, starvation, ischemia	ATG machinery; Beclin‐1; Na^+^/K^+^‐ATPase	Perinuclear ballooning; ER dilation; strong adhesion	Context‐dependent; no classically inflammatory	Ischemia–reperfusion, viral infection, cancer	[38]
Cuproptosis	Copper overload + mitochondrial respiration	FDX1; lipoylated TCA enzymes (DLAT); Fe–S proteins	Mitochondrial proteotoxic stress	Under investigation	Cancer vulnerabilities; copper tox	[46]
Entosis	Cell competition detachment, glucose starvation	RHOA–ROCK; cadherins; actomyosin	Cell‐in‐cell structures; lysosomal degradation	Indirect immune effects; context‐dependent	Tumor evolution aneuploidy	[55]
Ferroptosis	Iron‐dependent lipid peroxidation	System Xc‐GSH, GPX4; ACSL4; FSP1	Shrunken mitochondria; membrane damage	Often immunogenic via DAMPs	Cancer, neurodegeneration, I/R injury	[61, 62, 63]
Lysosomal cell death	Lysosomal membrane permeabilization/ rupture	Cathepsins (B/L/D); ROS; Hsp70	From apoptosis‐like to necrotic rupture	Can amplify inflammation; inflammasome priming	Cancer therapy, neurodegeneration, LSDs	[73, 74]
Mitotic catastrophe	Mitotic failure, DNA damage, spindle defects	p53, SAC proteins, Aurora kinases; CDK1	Multinucleation; micronuclei	Can be immunogenetic depending on outcome	Cancer therapy response, CIN	[79, 80]
Necroptosis	Death receptors/TLRs with Caspase‐8 blockade	RIPK1/RIPK3 → MLKL	Swelling; membrane rupture	Strongly inflammatory	Infection, I/R injury, neuroinflammation	[89]
NETosis	Neutrophils activation; infection; sterile triggers	Pad4, NE, MPO; ROS; sometimes GSDMD	NET release; lytic or vital	Proinflammatory; immunothrombosis	Sepsis, thrombosis, autoimmunity, cancer	[100]
PANoptosis	Pathogen/cytokine‐driven	PANoptosome: ZBP1, Caspase‐8, RPK3, ASC, gasdermins	Mixed features of apoptosis/pyroptosis/necroptosis	Highly inflammatory	Infection, autoinflammation, cancer	[105, 106]
Parthanatos	PARP‐1 hyperactivation after DNA damage	PARP‐1, PAR, AIF, NAD^+^ depletion	Large DNA fragmentation; metabolic collapse	Inflammatory via DAMPs	Stroke, neurodegeneration, diabetes, cancer	[111]
Pyroptosis	Inflammasome; cytosolic LPS; Caspase‐3/GSDME; granzymes	Caspase‐1/4/5/11; GSDMD/E/B; IL‐1/18	Cell swelling; gasdermin pore; lysis	Strongly inflammatory	Sepsis, inflammatory disease, cancer therapy	[114, 115]

### 2.14 Crosstalk Between Pyroptosis and Other Forms of PCD

Although pyroptosis was initially described as a distinct and autonomous form of inflammatory cell death, accumulating evidence now supports the concept that pyroptosis is deeply interconnected with other PCD modalities through shared molecular regulators, overlapping signaling pathways, and context‐dependent switches. Rather than functioning as an isolated endpoint, pyroptosis frequently emerges as part of a broader cell death network, in which apoptotic, necroptotic, and autophagy‐related signals converge to shape cellular fate and immune outcomes.

One of the most prominent examples of this crosstalk is the intimate relationship between pyroptosis and apoptosis. Caspase‐3, classically regarded as an executioner of apoptosis, can cleave gasdermin E (GSDME), converting a noninflammatory apoptotic program into a lytic and inflammatory pyroptotic response. This apoptotic‐to‐pyroptotic switch has been extensively documented in cancer cells exposed to chemotherapeutic agents and radiotherapy, where high GSDME expression transforms Caspase‐4 activation into membrane pore formation and secondary inflammatory signaling. This mechanism determines whether caspase activation results in silent cell clearance or inflammatory cell death. Conversely, in cells lacking functional gasdermins, pyroptotic stimuli may be rerouted toward apoptotic or necrotic outcomes, enhancing the plasticity of death execution.

Pyroptosis also exhibits substantial overlap with necroptosis, particularly at the level of inflammatory signaling and membrane disruption. Both processes culminate in plasma membrane rupture and the release of DAMPs, yet they are initiated by distinct upstream regulators. Importantly, several studies demonstrate that inhibition of Caspase‐8, a central checkpoint between apoptosis and necroptosis, can simultaneously promote necroptotic signaling via RIPK1/RIPK3 and inflammasome‐driven pyroptosis. This convergence is further exemplified in PANoptosis, where inflammasome components (ASC, Caspase‐1), apoptotic caspases (Caspase‐8), and necroptotic kinases (RIPK3) assemble into a single supramolecular complex. In this integrated framework, pyroptosis acts not merely as an endpoint but as a cooperative amplifier of inflammatory death programs during infection, cytokine storm, and tumor immune surveillance.

Autophagy‐dependent pathways may also modulate pyroptotic signaling in a bidirectional manner. Basal autophagy often suppresses excessive inflammasome activation by removing damaged mitochondria and limiting mitochondrial ROS production, thereby restraining pyroptosis. However, defective or overwhelmed autophagy can potentiate pyroptosis by promoting lysosomal destabilization, NLRP3 activation, and gasdermin‐mediated pore formation. In this sense, autophagy serves as a rheostat that determines whether cells undergo adaptive survival, autosis, or inflammatory pyroptotic death. LMP further strengthens that connection, as cathepsin release has been shown to trigger inflammasome activation upstream of pyroptosis in multiple pathological contexts.

Emerging evidence also links pyroptosis with metabolically driven death programs such as ferroptosis and cuproptosis. Oxidative stress, lipid peroxidation, and mitochondrial dysfunction, hallmarks of ferroptosis, can prime inflammasome activation and sensitize cells to pyroptotic execution. Conversely, pyroptotic membrane rupture may exacerbate redox imbalance and iron dysregulation, creating a feed‐forward loop that reinforces ferroptotic damage. While cuproptosis remains less explored in this context, copper‐induced mitochondrial stress and proteotoxicity may indirectly intersect with inflammasome signaling through mitochondrial danger signals, suggesting potential mechanistic overlap that warrants further investigation.

Collectively, these findings support a paradigm shift in which pyroptosis is best understood not as a solitary cell death modality, but as a dynamic node within an interconnected regulated cell death network. Its ability to intersect with apoptosis, necroptosis, autophagy‐dependent death, and metabolic stress responses positions pyroptosis as a decisive determinant of inflammatory tone, immune activation, and disease progression. Appreciating this crosstalk is therefore essential for interpreting experimental outcomes and for designing therapeutic strategies that aim to modulate cell death without inadvertently amplifying pathological inflammation or immune dysregulation.

This crosstalk framework helps explain why pyroptosis exerts divergent effects across diseases and therapeutic target settings, reconciling its pathogenic role in chronic inflammation with its beneficial exploitation in cancer therapy.

Table 2 highlights the differences between pyroptosis and the other PCDs. Finally, these PCD modalities illustrate the complexity and plasticity of cellular fate decisions. Rather than operating as isolated mechanisms, apoptosis, necroptosis, ferroptosis, autophagy‐dependent cell death, and related pathways form an integrated network regulated by shared molecular components and context‐dependent signals. This conceptual framework provides the necessary foundation for understanding pyroptosis as a specialized yet interconnected form of regulated cell death, whose unique inflammatory features and molecular execution are examined in detail in the following sections.

**TABLE 2:** Distinctive features of pyroptosis compared with other forms of programmed cell death.

Programmed cell death	Primary triggers(s)	Core molecular mediators	Plasma membrane integrity	Inflammatory profile	Key distinction compared with pyroptosis	References
Pyroptosis	PAMPs/DAMPs, cytosolic LPS, inflammasome activation, immune cytotoxic signals	Caspase‐1, Caspases‐4/5/11, Caspase‐3 (GSDME), granzymes A/B, gasdermins (GSDMD/E/B), inflammasomes, (NLRP3, AIM2), ASC	Lost (gasdermin pore formation)	Strongly proinflammatory (IL‐1β, IL‐18, DAMPs)	Lytic, gasdermin‐mediated death that couples cell elimination with potent immune activation	[114, 115]
Apoptosis	DNA damage, growth factor withdrawal, death receptor signaling	Caspase‐8, Caspase‐9, Caspase‐3/7, BCL‐2 family	Preserved	Immunologically silent or tolerogenic	Nonlytic, anti‐inflammatory; lacks cytokine release and membrane pore formation	[15, 16]
Anoikis	Loss of ECM attachment	Integrins, FAK, PI·K/AKT, Caspase‐8/9	Preserved	Silent	Context‐dependent apoptotic subtype; unrelated to inflammasome or innate immunity	[27, 28]
Autosis	Excessive or dysregulated autophagy	Beclin‐1, ATG proteins, Na^+^/K^+^‐ATPase	Preserved	Low or context‐dependent	Driven by autophagic flux, not inflammatory caspases or gasdermins	[38]
Cuproptosis	Intracellular copper overload	FDX1, lipoylated TCA enzymes (DLAT)m Fe–S proteins	Preserved	Poorly defined	Metabolic mitochondrial proteotoxic stress without immune cytokine signaling	[46]
Entosis	Cell–cell competition, detachment, mechanical stress	RHOA–ROCK, actin–myosin, cadherins, lysosomes	Preserved (host cells)	Low	Nonautonomous death via cell‐in‐cell invasion, not self‐executed lysis	[55]
Ferroptosis	Lipid peroxidation, iron accumulation, GPX4 inhibition	Fe^2+^, ROS, GPX4, SLC7A11, PUFAs	Lost (late stage)	Moderately inflammatory	Oxidative, noncaspase death; lacks gasdermin pores and IL‐1β/ IL‐18 maturation	[61, 62, 63]
Lysosomal cell death	Lysosomal membrane permeabilization	Cathepsins (B, D, L), ROS, Hsp70	Variable	Proinflammatory (indirect)	Initiated by lysosomal rupture rather than inflammasome–gasdermin axis	[73, 74]
Mitotic catastrophe	DNA damage, spindle defects, checkpoint failure	p53, CDK1‐cyclin B, Aurora kinases	Variable	Context‐dependent	Prelethal process that culminates in apoptosis/necroptosis, not a primary inflammatory death	[79, 80]
Necroptosis	Death receptors PRRs when Caspase‐8 is inhibited	RIPK1, RIPK3, MLKL	Lost (MLKL pores)	Strongly proinflammatory	Lytic like pyroptosis but caspase‐independent and gasdermin‐independent	[89]
NETosis	Pathogens sensing, ROS immune activation (neutrophils)	PAD4, NE, MPO, ROS, GSDMD	Lost or preserved (vital NETosis)	Proinflammatory	Restricted to neutrophils; specialized antimicrobial function rather than general PCD	[100]
PANoptosis	Infection, cytokine storm, cellular stress	PANoptosome (CASP8, RIPK3, ASC; ZBP1), caspases, gasdermins	Lost	Highly proinflammatory	Integrates pyroptosis, apoptosis and necroptosis; pyroptosis acts as one component	[105, 106]
Parthanatos	Excessive DNA damage	PARP‐1, PAR polymers, AIF	Lost (Late)	Proinflammatory	Caspase‐independent nuclear catastrophe; no gasdermin pores or cytokine maturation	[111]

## 3 The Molecular Pathway of Pyroptosis

Pyroptosis is a tightly orchestrated inflammatory cell death program that connects danger sensing to membrane rupture and cytokine release. In this section, a structured, pyroptosis‐centered overview of how inflammasome activation and inflammatory caspases converge on gasdermin cleavage to form membrane pores is provided, distinguishing canonical and noncanonical routes while highlighting alternative entry points and regulatory layers. This mechanistic framework is necessary to interpret how distinct upstream stimuli, molecular checkpoints, and executioner gasdermins shape downstream inflammatory outputs and tissue consequences.

### 3.1 Canonical Pathway

The innate immune system constitutes the organism's immediate defense barrier, responsible for detecting and responding to microbial invasion and endogenous stress signals [119]. It relies on pattern recognition receptors (PRRs), which identify conserved molecular motifs known as pathogen‐associated molecular patterns (PAMPs) and DAMPs [120, 121]. Importantly, the sensitivity of this system is shaped by upstream priming signals derived from TLRs, cytokine receptors, and metabolic cues, which regulate the expression levels and activation threshold of inflammasome components [122].

Upon detection of PAMPs and DAMPs, intracellular PRRs such as nucleotide‐binding domain and leucine‐rich repeat‐containing receptors (NLRs) or AIM2‐like receptors (ALRs) undergo oligomerization, recruiting adaptor and effector proteins that culminate in the assembly of multiprotein signaling complexes collectively termed inflammasomes [123, 124]. This activation step is highly context‐dependent and varies across cell types; for instance, professional immune cells such as macrophages exhibit rapid inflammasome assembly, whereas epithelial cells often display delayed or attenuated activation kinetics [125].

In the canonical pathway, these sensors include the NLRs such as NLRP1, NLRP3, and NLRC4, as well as ALRs and pyrin [126, 127, 128, 129, 130, 131, 132, 133]. Upon recognition of their respective stimuli, these sensor proteins undergo conformational changes and oligomerization mediated by their NACHT domains. Oligomerized sensors recruit the adaptor protein ASC (apoptosis‐associated speck‐like proteins containing a CARD) through homotypic PYD–PYD interactions [134]. ASC subsequently serves as a molecular bridge by engaging procaspase‐1 via CARD–CARD interactions, facilitating proximity‐induced autocatalytic cleavage and activation of Caspase‐1 [135].

Activated Caspase‐1 functions as the central executioner of canonical pyroptosis. It cleaves GSDMD, releasing an N‐terminal fragment that oligomerizes within the plasma membrane to form large, nonselective pores [136]. These pores disrupt ionic homeostasis, induce osmotic swelling, and culminate in membrane rupture, which constitutes the morphological hallmark of pyroptotic cell death [137]. In parallel, Caspase‐1 processes the inactive precursor of IL‐1β and IL‐18 into their mature forms, which are released through GSDMD pores to amplify local and systemic inflammation [138].

This tightly regulated cascade ensures that danger sensing by specific inflammasome sensors is directly coupled to both cytokine release and inflammatory cell death, enabling an efficient innate immune response (Tables 3 and 4).

**TABLE 3:** Principal inflammasome molecules involved in the canonical pyroptosis pathway.

Inflammasome/molecule	Main expression sites	Core components	Activation triggers/signals	Molecular mechanism	Functional outcomes	Relevance in disease and inflammation	References
NLRP1 inflammasome	Epithelial and hematopoietic cells	NLRP1 sensor, ASC adaptor, and procascaspe‐1	Cellular stress, toxins, microbial signals	Danger recognition induces NLRP1 oligomerization, ASC recruitment, and Caspase‐1 activation, leading to GSDMD cleavage and cytokine maturation.	Induces IL‐1β/ IL‐18 release and pyroptotic membrane rupture	NLRP1 dysregulation aggravates colitis and Th1 inflammation; inhibition reduces IL‐1β, IL‐18, and TNF‐α in IBD models.	[130]
NLRP3 inflammasome	Macrophages, dendritic cells, epithelial cells	NLRP3 receptor, ASC, and procaspase‐1	Priming via (TLR4, ILβ, TNF‐α) + ATP, ROS, toxins	NF‐κB‐dependent priming followed by ion‐flux driven oligomerization and ASC recruitment	Caspases‐1 activation, IL‐1β/IL‐18 maturation, an GSDMD cleavage	Central driver of IBD, neuroinflammation, tumorigenesis	[129]
NLRC4 inflammasome	Macrophages, dendritic cells, intestinal epithelia	NLRC4 receptor, NAIP proteins, ASC, and procaspase‐1	Bacterial flagellin and components of Type III secretion systems (T3SS)	NAIP recognition of bacterial ligands trigger NLRC4 oligomerization, ASC recruitment, and Caspase‐1 activation.	Promotes pyroptosis and β/ IL‐18 secretion, restricting intracellular pathogens	Protects against bacterial invasion and tumor progression; NLRC4 deficiency enhances tumorigenesis in colitis‐associated cancer.	[131]
AIM2 inflammasome	Epithelial cells and macrophages in the small and large intestine	AIM2 receptor (ALR family), ASC adaptor, and procaspase‐1	Cytoplasmatic double‐stranded DNA (from pathogens, viruses, or damaged host cells)	DNA binding to the HIN‐200 domain releases AIM2 autoinhibition, exposing the PYD domain that recruits ASC and activates Caspase‐1.	Leads to β/IL‐18 maturation and pyroptosis; regulates intestinal immune homeostasis	AIM2 protects against DSS‐induced colitis and limits gut dysbiosis; its loss promotes inflammation and tumor growth; overexpression suppresses BRAF‐mutated CRCR through Caspase‐1 activation.	[132]
Pyrin inflammasome	Granulocytes. Monocytes, eosinophils, and dendritic cells	Pyrin sensor (encoded by *MEFV*), ASC adaptor, and procaspase‐1	Inactivation of Rho family GTPases or cytoskeletal disruption	The PYD domain of pyrin recruits ASC, forming the pyrin inflammasome that activates Caspase‐1 and promotes IL‐1β processing.	Mediates IL‐1β release and pyroptotic death in response to bacterial toxins affecting Rho GTPases	Mutations in pyrin's B30.2 domain cause familial Mediterranean fever (FMF); abnormal pyrin signaling contributes to autoinflammatory syndromes such as PAPA and FMF.	[133]
ASC/PYCARD (TMS1)	Epithelial cells, monocytes, macrophages, granulocytes, dendritic cells	Bipartite adaptor protein with N‐terminal PYD and C‐terminal CARD domains	Activated downstream of multiple inflammasome sensor (NLRP3, AIM2, pyrin); aggregation into cytosolic specks after activation	Serves as a bridging adaptor: PYD binds to inflammasome receptors (NLRs/ALRs) and CARD binds procaspase‐1; drives inflammasome assembly, Caspase‐1 activation, and IL‐1β/IL‐18 maturation; also forms extracellular ASC specks acting as DAMPs	Facilitates cytokine processing, pyroptosis, and inflammation; can also induce apoptosis in certain contexts	Dual role in cancer; acts as a tumor suppressor via apoptosis induction and promoter of tumor‐associated inflammation through IL‐1/IL‐18 signaling; aberrant methylation or overexpression alters outcomes in melanoma, gastric, and pancreatic cancer.	[134, 135]

**TABLE 4:** Functional comparison of caspases involved in pyroptosis.

Caspase	Pathway	Activation trigger/sensor	Adaptor proteins	Primary substrate(s)	Molecular function	Inflammatory output/cellular effect	Biological or pathophysiological context	References
Caspase‐1	Canonical	Activated through inflammasome complexes (NLRP1, NRLP3, NLRC4, AIM2, pyrin) sensing PAMPs or DAMPs	ASC (PYD–PYD and CARD–CARD interactions)	Gasdermin D, pro‐IL‐1β, pro‐IL‐18	Executes pyroptosis by cleaving GSDMD; activates inflammatory cytokines	Causes plasma membrane pore formation, IL‐1β and IL‐18 secretion, osmotic swelling and lytic death	Central effector of the canonical pyroptotic pathway; key driver of innate immune inflammation in infection, IBD, and cancer	[115, 139]
Caspase‐4 (human)	Noncanonical	Direct recognition of cytosolic LPS from Gram‐negative bacteria	None (direct LPS bonding via CARD domain)	Gasdermin D	Function as intracellular LPS sensor and GSDMD activator	Induces pyroptotic pore formation and K^+^ efflux, leading to secondary NLRP3 inflammasome activation	Defends against intracellular bacterial infection; contributes to septic inflammation when overactivated	[140]
Caspase‐5 (human)	Noncanonical	Cytoplasmic LPS or infection‐derived signals (cooperated with Caspase‐4)	None (CARD‐dependent oligomerization)	Gasdermin D	Acts synergistically with Caspase‐4 to cleave GSDMD	Promotes pyroptotic lysis and amplifies inflammatory cytokine production via NLRP3 engagement	Upregulated in monocytes during endotoxemia and chronic inflammatory disorders	[140]
Caspase‐11 (murine ortholog of human Caspase‐4/5)	Noncanonical	Recognition of intracellular LPs from Gram‐negative bacteria	None	Gasdermin D	Triggers noncanonical pyroptosis in mice	Drives membrane rupture and secondary activation of Caspase‐1/NLRP3	Critical for antibacterial defense; excessive activation leads to lethal endotoxemia	[140]
Caspase‐3	Alternative (GSDME‐mediated)	Activated during apoptosis (extrinsic or intrinsic)	—	Gasdermin E	Converts apoptosis into pyroptosis by cleaving GSDME	Induces secondary pyroptotic membrane rupture in tumor or stressed cells	Relevant un cancer therapy induced inflammatory death; connects apoptosis and pyroptosis	[141, 142]
Caspase‐8	Alternative/ cross‐regulated	Engaged under conditions of apoptosis inhibition, viral infection, or TLR stimulation	FADD, ASC (in certain cell types)	Gasdermin D, pro‐IL‐1β	Acts as a bridge between apoptosis and pyroptosis	Induces pyroptosis when apoptotic pathways are blocked; may also promote IL‐1β maturation	Links inflammasome signaling to apoptotic regulation and PANoptosis	[143, 144, 145, 146]

### 3.2 Noncanonical Pathway

Noncanonical pyroptosis represents a distinct inflammatory cell death pathway triggered by the direct detection of intracellular LPSs derived from Gram‐negative bacteria by inflammatory caspases [147, 148]. In humans, this function is mediated by Caspases‐4 and ‐5, whereas Caspase‐11 serves as their functional ortholog in mice (Table 4) [140]. Instead of relying on classical pattern‐recognition receptors, these caspases function as intracellular sensors whose activation depends on the efficient delivery of LPS to the cytosol, a process regulated by host factors that control bacterial access and membrane integrity [149].

Upon binding to LPS through their CARD domains [150], Caspase‐4/5/11 oligomerize and autoactivate, initiating the proteolytic cleavage of GSDMD [151]. The liberated N‐terminal fragment of GSDMD inserts into the plasma membrane and forms pores [152]. Similar to the canonical pathway, pore formation can be temporally graded: early sublytic pores promote potassium efflux without immediate cell lysis, whereas prolonged pore activity leads to catastrophic membrane rupture [153].

Although noncanonical caspases do not directly activate proinflammatory cytokines, the potassium efflux induced by GSDMD pores serves as a secondary signal that activates the NLRP3 inflammasome, leading to Caspase‐1 activation and subsequent maturation of IL‐1β and IL‐18 [115, 139]. Accessory molecules further amplify this pathway. Guanylate‐binding proteins promote cytosolic sensing of bacteria, while pannexin‐1 cleavage, ATP release, and carrier‐mediated LPS transport via HMGB1 or SCGB3A2 fine‐tune signal intensity and inflammatory outcome [154, 155, 156]. The balance between sublytic signaling and terminal lysis is influenced by cell type and gasdermin expression levels, ensuring that noncanonical pyroptosis can function both as an inflammatory amplifier and as a terminal antibacterial defense mechanism.

### 3.3 Caspase‐3/8‐Mediated Pathway

Emerging evidence indicates that pyroptosis is not exclusively dependent on Caspase‐1 or ‐11 activation but can also be initiated through specific apoptotic caspases under defined physiological or pathological contexts [157, 158]. In these settings, the critical switch is the proteolytic activation of specific gasdermin family members, which redirects classical apoptotic signaling toward plasma membrane pore formation and inflammatory lysis [159].

Caspase‐3, traditionally considered a core effector of apoptosis, can induce pyroptosis by cleaving GSDME [160]. This cleavage releases the pore‐forming N‐terminal domain of GSDME, which oligomerizes in the plasma membrane and triggers secondary pyroptosis, particularly following cytotoxic stress or chemotherapy‐induced apoptosis‐like programs [141, 142]. Therefore, Caspase‐3 does not “replace” inflammasomes, but rather provides an alternative upstream entry point that converges in the same terminal event: gasdermin‐mediated membrane permeabilization.

Likewise, Caspase‐8 functions as a molecular hub connecting inflammatory receptor signaling and cell death decisions [161]. Under conditions where apoptosis is restrained or when pathogen factors interfere with prosurvival checkpoints (e.g., TAK1 inhibition), Caspase‐8 can assemble in death‐inducing platforms (such as RIPK1–FADD–Caspase‐8 complexes) and cleave GSDMD (and, depending on context, GSDME), thereby initiating pore formation and pyroptotic lysis [143, 144, 145, 146] (Table 4). Beyond GSDMD/GSDME, Caspase‐8‐mediated activation of gasdermin C has been described in tumor contexts driven by PD‐L1 and TNF‐α signaling, enhancing an additional route by which inflammatory cues can engage gasdermin execution and inflammatory cell death [137, 162, 163].

Together, these pathways expand the mechanistic framework of pyroptosis by demonstrating that distinct upstream caspases can converge on gasdermin activation to produce a pyroptotic endpoint, reinforcing the concept of modular, context‐dependent inflammatory cell death programs

### 3.4 Functional Crosstalk Between Gasdermin Isoforms and Pyroptotic Signaling Pathways

Members of the gasdermin family execute pyroptotic cell death in a highly context‐dependent manner because their expression in tissue‐biased cells and their activation depend on distinct proteases [164]. Conceptually, gasdermins act as “execution modules”: once a compatible protease cleaves the inhibitory C‐terminal domain, the liberated N‐terminal fragment becomes competent to insert into membranes and assemble pores, thereby converting upstream immune or stress signals into inflammatory membrane rupture [165].

GSDMA is enriched in epithelial barriers, including the upper gastrointestinal tract and skin, where it can be activated by bacterial proteases. For example, SpeB cleavage releases an N‐terminal fragment that forms a membrane pore and promotes pyroptosis in keratinocytes, thereby contributing to restriction of bacterial replication at barrier sites [166, 167, 168].

GSDMB, primarily expressed in human epithelial tissues, can be processed by granzyme A, and specific isoforms have also been reported to be cleaved by Caspase‐1, enabling pore formation and pyroptosis in epithelial and tumor cells, particularly in immune effector contexts [169, 170, 171].

In tumor microenvironments, GSDMC expression is frequently elevated and may be engaged downstream of TNF‐α signaling via Caspase‐8, or through ketoglutarate‐associated signaling platforms, again illustrating how inflammatory cues can be rewired into gasdermin‐driven membrane permeabilization [172, 173].

GSDME, broadly expressed across tissues, provides a direct mechanistic bridge between apoptosis and pyroptosis by being cleaved by Caspase‐3 or granzyme B (GzmB), which can amplify immune‐mediated inflammatory killing and reshape the immunogenicity of dying cells [174, 175].

Finally, DFNB59 retains structural features related to gasdermins but lacks canonical pore‐forming pyroptotic activity, illustrating functional diversification within the family and emphasizing that not all gasdermin‐like proteins operate as classical executioners [176, 177].

Collectively, these isoform‐specific activation routes explain how distinct protease environments (in infection, cytotoxic immunity, or tumor signaling) determine which gasdermin is engaged and how strongly pyroptotic outcomes are expressed (Table 5).

**TABLE 5:** Key gasdermin family members, activators, and their roles in pyroptosis.

Gasdermin	Expression	Activators	Cleavage site	Pyroptotic outcome/function	Reference
GSDMA	Upper gastrointestinal tract, skin epithelium	Bacterial protease SpeB	Gln246	Barrier‐associated executioner: SpeB‐mediated cleavage releases pore‐forming N‐terminus → plasma membrane permeabilization → pyroptosis in keratinocytes; contributes to restriction of bacterial replication	[166, 167, 168]
GSDMB	Epithelial tissues (gastrointestinal. respiratory)	Granzyme A, Caspase‐1 (certain isoforms)	Lys244 (GZMA), Asp236 (Caspase‐1)	Cytotoxic immunity/tissue contexts: N‐terminal liberation → pore formation → pyroptosis in epithelial and cancer cells; can amplify inflammatory signaling depending on isoform and protease availability	[169, 170, 171]
GSDMC	Various tissues; upregulated in tumors	Caspsae‐8 (TNF‐α pathway), Caspase‐8 (ketoglutarate/receptosome pathway)	Asp365 (TNF‐α), Asp240 (KG)	Tumor/inflammatory signaling module: Caspase‐8 cleavage → pore‐forming N‐terminus → membrane perforation → pyroptosis; strongly context‐dependent	[172, 173]
GSDME	Broad tissue expression (GI tract, brain, uterus, smooth muscle)	Caspase‐3, granzyme B	Asp270	Apoptosis‐to‐pyroptosis switch: Caspase‐3 cleavage (or granzyme B) releases pore‐forming N‐terminus → secondary pyroptosis; increases immunogenic/inflammatory features of cell death	[174, 175]
DNFB59	Auditory system	—	—	Gasdermin‐like features but noncanonical/nonpyroptotic activity; functional role remains under investigation	[176, 177]

**FIGURE 2:**
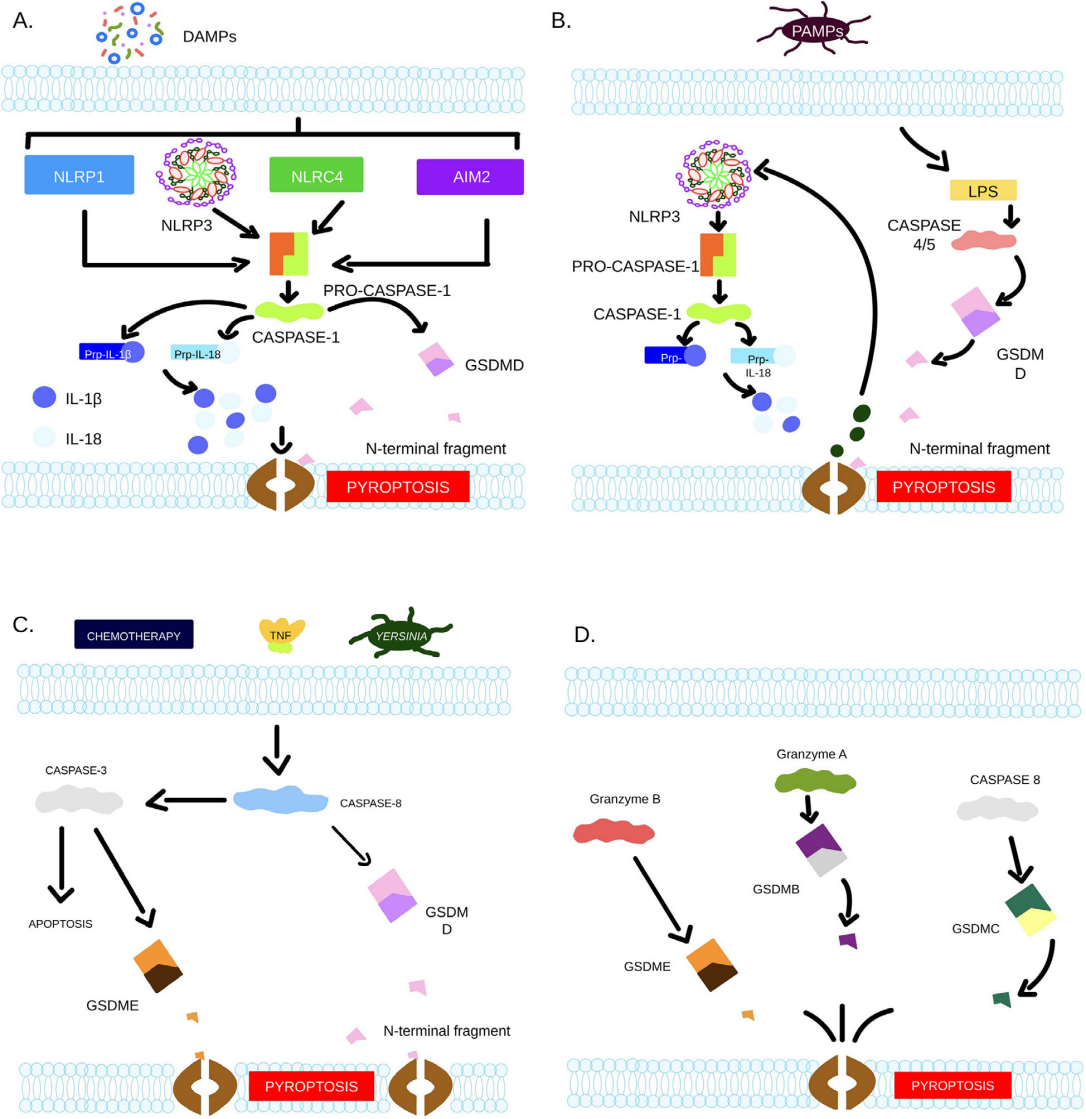
Canonical, noncanonical, caspase‐dependent, and granzyme‐mediated pathways converge on gasdermin activation to execute pyroptotic cell death. (A) DAMP sensing by inflammasome receptors (NLRP1, NLRP3, NLRC4, AIM2) activates caspase‐1, leading to IL‐1β/IL‐18 maturation and GSDMD‐mediated membrane pore formation. (B) Intracellular LPS activates caspase‐4/5 (caspase‐11 in mice), inducing GSDMD cleavage and pyroptosis, with parallel amplification through NLRP3 inflammasome signaling. (C) Apoptotic or stress stimuli engage Caspase‐8 or Caspase‐3 to cleave GSDMD or GSDME, redirecting apoptosis toward inflammatory pyroptosis. (D) Cytotoxic lymphocyte‐derived granzymes cleave gasdermins (GSDMB, GSDME) directly or via Caspase‐3, resulting in pore formation and pyroptotic cell death.

Collectively, the pathways detailed above illustrate that pyroptosis is not a single linear cascade but a modular network in which inflammasomes, caspases, and gasdermins act as interchangeable nodes that determine the intensity and quality of the inflammatory outcome. The diversity in gasdermin expression and activation routes (e.g., tissue‐enriched GSDMA/GSDMB and context‐dependent GSDMC/GSDME engagement) provides an additional layer of specificity that can shift pyroptosis toward host protection or pathology. Building on this mechanistic foundation, the next section links these molecular circuits to disease settings in which pyroptosis becomes either a driver of immunopathology or a tractable therapeutic axis. All of these pathways are linked in Figure 2.

## 4 Pyroptosis in Human Disease: Mechanisms and Pathophysiological Implications

Pyroptosis is increasingly recognized as a context‐dependent disease modifier: protective when it eliminates infected or transformed cells, yet harmful when excessive activation sustains cytokine amplification and tissue injury. Here, it is integrated mechanistic principles, such as inflammasome sensing, inflammatory caspase activation, and gasdermin‐driven pore formation, with organ‐ and cell‐type‐specific consequences across infections, inflammatory/autoimmune conditions, metabolic‐cardiovascular disorders, and cancer. This organization aims to clarify how shared core modules generate divergent pathophysiological outputs depending on stimulus duration, cellular compartmentalization, and the surrounding immune microenvironment.

### 4.1 Pyroptosis in Microbial Infections: Molecular Players and Immunopathological Impact

Pyroptosis plays a pivotal role in shaping host–pathogen interactions during bacterial, viral, and fungal infections, serving both as a protective mechanism and a potential source of immunopathology [178]. *Salmonella enterica* initiates pyroptosis through the activation of NLRC4 and NLRP3 inflammasomes upon detection of flagellin and Type III secretion system components, leading to Caspase‐1‐mediated cleavage of GSDMD and release of IL‐1β and IL‐18 [179]. This process facilitates bacterial clearance by eliminating infected macrophages and epithelial cells [180]. Similarly, *Listeria monocytogenes* activates AIM2 and NLRP3 inflammasomes in macrophages through cytosolic DNA sensing, resulting in GSDMD‐dependent pyroptosis that enhances immune elimination [181]. In the intestinal tract, pathogenic *Escherichia coli* strains engage the noncanonical pathway involving Caspases‐4, ‐5, and ‐11 in response to cytosolic LPS, leading to epithelial pyroptosis that promotes mucosal inflammation and bacterial expulsion [181].

In tuberculosis, *Mycobacterium tuberculosis* triggers NLRP‐3‐driven pyroptosis in macrophages, a process that contributes to granuloma formation while simultaneously facilitating bacterial dissemination through cell lysis [183, 184]. *Yersinia* species employ the virulence effector YopJ to inhibit TAK1 signaling and activate the RIPK1–FADD–Caspase‐8 complex, thereby inducing GSDMD‐ and GSDME‐mediated pyroptosis that reinforces innate immune activation [185, 186]. In contrast, *Streptococcus pyogenes* elicits a distinct form of pyroptosis through direct cleavage of GSDMA by its secreted cysteine protease SpeB in keratinocytes, serving as a barrier defense mechanism against localized infection [187]. Likewise, *Shigella flexneri* activates the NAIP–NLRC4 inflammasome in macrophages, triggering Caspase‐1‐dependent pyroptosis that limits intracellular bacterial proliferation [188, 189].

Viral pathogens also harness or activate pyroptotic pathways as part of the host response. *Influenza A virus* (*IAV*) stimulates NLRP3 inflammasome activation in alveolar macrophages and epithelial cells, inducing cytokine release that supports antiviral defense but, when excessive, provokes tissue injury and lung inflammation [190, 191]. *SARS‐COV‐2*, the causative agent of COVID‐19, engages both NLRP3 and AIM22 inflammasome in monocytes, endothelial and epithelial cells, leading to Caspase‐1 activation, GSDMD cleavage, and the hypersecretion of IL‐1β and IL‐18 [192]. This hyperinflammatory state contributes to the cytokine storm and acute lung pathology seen in severe infections [193]. Meanwhile, *HIV‐1* triggers pyroptosis in abortively infected CD4^+^T cells via an IFI‐16 and Caspase‐1‐dependent mechanism, resulting in progressive T‐cell depletion and chronic immune activation [194].

Importantly, fungal pathogens have also been linked to pyroptotic signaling. *Candida albicans* activates the NLRP3 inflammasome in macrophages and dendritic cells through recognition of β‐glucans and other fungal components, leading to Caspase‐1‐mediated cleavage of GSDMD and subsequent IL‐1β and IL‐18 release [195, 196]. This response enhances antifungal immunity by promoting the recruitment of neutrophils and the elimination of fungal cells; however, excessive or sustained activation can exacerbate tissue damage and inflammation [197].

Overall, in infectious diseases, pyroptosis functions primarily as a pathogen‐sensing and host‐defense mechanism, eliminating infected cells and amplifying innate and adaptive immune responses. However, when pyroptosis is excessive or abortive, as observed in HIV infection, it leads to massive immune cell loss and persistent immune activation, thereby contributing to disease progression rather than pathogen clearance (Table 6).

**TABLE 6:** Pyroptosis mechanism in infectious diseases: Inflammasome activation and host responses.

Pathogen/infectious disease	Primary inflammasome or activation pathway	Key molecular mediators (caspases/gasdermins)	Main cellular targets	Pathophysiological consequences	References
*Salmonella enterica*	NLRC4 and NLRP3 inflammasomes	Caspase‐1, GSDMD, IL‐1β, IL‐18	Macrophages, epithelial cells	Promotes pyroptotic clearance of infected cells; limits bacterial replication and enhances inflammation	[179]
*Listeria monocytogenes*	AIM2 and NLRP3 inflammasomes	Caspase‐1, GSDMD	Macrophages	Cytosolic bacterial DNA recognition induces pyroptosis, enhancing bacterial elimination	[181]
*Escherichia coli* (pathogenic strains)	Noncanonical inflammasomes (Caspase‐4/‐5/‐11)	Caspase‐4/‐5/‐11, GSDMD	Intestinal epithelial cells	Cytosolic LPS detection induces pyroptosis‐contributing to mucosal inflammation and bacterial expulsion	[182]
*Mycobacterium tuberculosis*	NLRP3 inflammasome	Caspase‐1, GSDMD	Macrophages	Pyroptosis supports granuloma formation but may facilitate bacterial dissemination	[183, 184]
*Yersinia spp*.	RIPK1–FADD–Caspase‐8 signaling (TAK1 inhibition)	Caspase‐8, GSDMD, GSDME	Macrophages	YopJ effector triggers Caspase‐8‐mediated pyroptosis, reinforcing innate immune activation	[185, 186]
*Streptococcus pyogenes*	Bacterial protease‐mediated activation	GSDMA	Keratinocytes	SpeB protease cleaves GSDMA to induce pyroptosis, restricting local bacterial proliferation	[187]
*Shigella flexneri*	NAIP–NLRC4 inflammasome	Caspase‐1, GSDMD	Macrophages	Activation of inflammasome and pyroptosis limits intracellular bacterial proliferation	[188, 189]
*Influenza A virus* (IAV)	NLRP3 inflammasome	Caspase‐1, GSDMD, IL‐1β, IL‐18	Alveolar macrophages, epithelial cells	Antiviral defense through cytokine release; excessive activation leads to lung inflammation	[190, 191]
*SARS‐CoV‐2* (COVID‐19)	NLRP3 and AIM2 inflammasomes	Caspase‐1, GSDMD, IL‐1β, IL‐18	Monocytes, endothelial and epithelial cells	Overactivation induces cytokine storm and severe pulmonary injury	[192]
*HIV‐1*	IFI16–Caspase‐1 pathway	Caspase‐1, GSDMD	CD4^+^ T cells	Abortive infection triggers pyroptosis, causing T‐cell depletion and chronic inflammation	[194]
*Candida albicans*	NLRP3 inflammasome	Caspase‐1, GSDMD, IL‐1β, IL‐18	Macrophages, dendritic cells	Recognition of fungal β‐glucans triggers pyroptosis, promoting antifungal immunity but potentially driving tissue damage	[195, 196]

### 4.2 Pyroptosis in Autoimmune and Autoinflammatory Diseases: Mechanisms of Dysregulated Inflammation and Tissue Damage

Pyroptosis has emerged as a pivotal process linking dysregulated innate immunity to chronic inflammation injury in autoimmune and autoinflammatory disorders [200]. In these conditions, excessive activation of inflammasomes, mainly NLRP3, AIM2, and pyrin, leads to Caspase‐1‐mediated cleavage of GSDMD, promoting pore formation, cytokine release, and cellular rupture [198, 199].

In systematic lupus erythematosus (SLE), monocytes and keratinocytes undergo GDMD‐dependent pyroptosis, releasing nuclear autoantigens and IL‐1β/IL‐18, which intensify autoreactive T and B cell activation [200, 201, 202]. Rheumatoid arthritis exhibits NLRP3 and P2×7 upregulation in synovial tissue, where pyroptotic fibroblast‐like synoviocytes contribute to pannus formation and cartilage erosion [203, 204]. In inflammatory bowel disease (IBD), epithelial cell pyroptosis driven by NEK7–GSDMD signaling disrupts barrier integrity, exacerbating mucosal inflammation [173, 205].

Similarly, Type 1 diabetes features β‐cell pyroptosis triggered by AIM2 and NLRP3 activation, amplifying local immune infiltration and accelerating insulin deficiency [206, 207]. Multiple sclerosis (MS) involves microglial and astrocytic inflammasome activation, leading to IL‐1β‐driven demyelination and neuroinflammation [208, 209]. In psoriasis, keratinocyte pyroptosis mediated by Caspase‐1 and GSDME fosters chronic IL‐17 and IL‐23 signaling loops, sustaining epidermal hyperplasia [210, 211].

Autoinflammatory syndromes such as familial Mediterranean fever (FMF) and TRAPS arise from germline mutations that constitutively activate inflammasomes, resulting in recurrent systemic inflammation [212, 213]. In pulmonary contexts, pneumococci, especially in bacterial and viral infections, can trigger exaggerated inflammasome activation in alveolar macrophages, promoting tissue damage through excessive pyroptotic cell death [214, 215].

In autoimmune and autoinflammatory disorders, pyroptosis is predominantly driven by dysregulated inflammasome activation rather than microbial sensing. Sustained gasdermin‐mediated cell death and chronic IL‐1β/IL‐18 release amplify self‐directed immune responses, promote tissue destruction, and maintain long‐lasting inflammatory loops that underlie disease chronicity (Table 7).

**TABLE 7:** Pyroptosis involvement in autoimmune and autoinflammatory diseases.

Disease	Principal inflammasome/pathway	Gasdermin/caspase mediators	Main cellular targets	Pathophysiological consequences	References
Systemic lupus erythematosus (SLE)	NLRP3, AIM2	Caspase‐1, GSDMD	Monocytes, keratinocytes	Cytokine release and nuclear antigen exposure amplify autoimmunity	[200, 201, 202]
Rheumatoid arthritis (RA)	NLRP3, P2×7	Caspase‐1, GSDMD	Synoviocytes, macrophages	Pyroptotic death drives synovial hyperplasia and joint erosion	[203, 204]
Inflammatory bowel disease (IBD)	NLRP3–NEK7 axis	Caspase‐1, GSDMD	Intestinal epithelial cells	Barrier loos and mucosal inflammation	[173, 205]
Type 1 diabetes	NLRP3, AIM2	Caspase‐1, GSDMD	Pancreatic β‐cells	Pyroptosis accelerates islet inflammation and insulin loss	[206, 207]
Multiple sclerosis	NLRP3, AIM2	Caspase‐1, GSDMD, GSDME	Microglia, astrocytes	Neuroinflammation and demyelination	[208, 209]
Psoriasis	Caspase‐1, NLRP1	GSDME, GSDMA	Keratinocytes	Epidermal pyroptosis promotes IL‐17/IL‐23 signaling	[210, 211]
Familial mediterranean fever (FMF)	Pyrin inflammasomes	Caspase‐1, GSDMD	Macrophages	Periodic IL‐1β release and systematic inflammation	[212, 213]
TRAPS	NLRP3 hyperactivation	Caspase‐1, GSDMD	Monocytes	Recurrent fever and cytokine excess	[214, 215]
Pneumonia (bacterial/viral)	NLRP3, AIM2	Caspase‐1, GSDMD	Alveolar macrophages, epithelial cells	Pyroptosis contributes to lung injury and cytokine storm	[214, 215]

### 4.3 Pyroptosis in Metabolic and Cardiovascular Disorders

Pyroptosis, a lytic form of PCD mediated primarily by gasdermins (GSDMD, GSDME, GSMDC), links cellular stress to chronic inflammation. In Type 2 diabetes mellitus, persistent hyperglycemia, insulin resistance, and β‐cell dysfunction trigger NLRP3 inflammasome activation, releasing IL‐1β and IL‐18, which amplify oxidative stress, NF‐κB signaling, and β‐cell death, worsening glucose homeostasis [216, 217, 218]. Obesity induces chronic meta‐inflammation in white adipose tissue, where hypertrophic adipocytes undergo pyroptosis, recruiting proinflammatory M1 macrophages and elevating IL‐1β/IL‐18, thereby promoting insulin resistance and metabolic comorbidities [219, 220, 221]. Metabolically associated fatty liver disease progresses from simple steatosis to steatohepatitis and fibrosis via hepatocyte, Kupffer cell, and stellate cell pyroptosis, driven by NLRP3/Caspase‐1/GSDMD signaling and DAMP‐mediated crosstalk [222, 223, 224].

In the cardiovascular system, pyroptosis contributes to myocardial infarction by exacerbating ischemia/reperfusion injury through ROS‐mediated NLRP3–GSDMD activation in cardiomyocyte death induced by hyperglycemia, high‐fat diet, and NLRP3/CMKLR1 axis activation, leading to impaired cardiac function [225, 226, 227]. Hypertension and pulmonary hypertension involve endothelial and smooth cell pyroptosis mediated by Caspase‐1/NLRP3, homocysteine, and LPS, contributing to vascular remodeling [228, 229, 230]. Other CVDs, including dilated cardiomyopathy, arrhythmia, myocarditis, and cardiac hypertrophy, are similarly linked to inflammasome‐driven pyroptosis, enhancing potent therapeutic targets across metabolic and cardiovascular diseases [231, 232, 233].

In metabolic and cardiovascular disease, pyroptosis arises mainly from prolonged metabolic, oxidative, or hemodynamic stress. Persistent inflammasome activation in parenchymal and vascular cells links cellular stress to low‐grade chronic inflammation, progressive tissue dysfunction, and the worsening of metabolic and cardiovascular pathology.

### 4.4 Pyroptosis in Cancer

**FIGURE 3:**
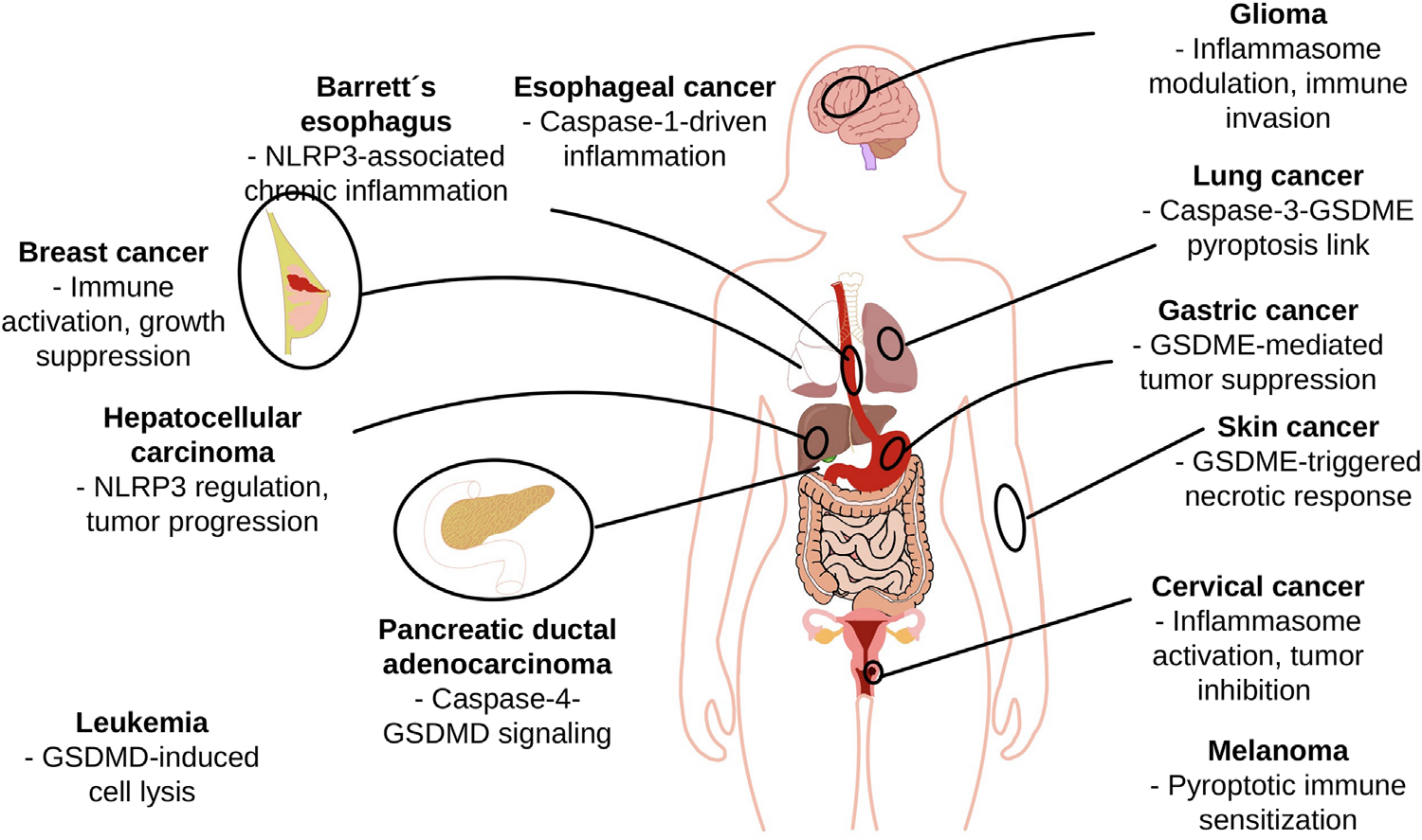
Context‐dependent roles of pyroptosis across different cancer types. Proptosis is involved in multiple cancers, where it can either promote tumor progression or enhance antitumor responses depending on cellular context and molecular regulation. Its broad involvement enhances pyroptotic pathways as potential therapeutic targets, while emphasizing the need for further investigation to translate these mechanisms into clinical benefit.

Cancer development is a complex process regulated by multiple factors, including the balance of oncogenes and tumor suppressor genes, persistent inflammation, oxidative stress, and the immune landscape of tissues [234]. Prolonged exposure of cells to inflammatory stimuli can elevate the likelihood of malignant transformation [235]. Pyroptosis, a lytic form of PCD, leads to the release of proinflammatory mediators such as IL‐1β and IL‐18, which may either facilitate or suppress tumor growth depending on the cellular context [236, 237]. Experiments in NLRP3−/− and Caspase‐1−/− mice demonstrate that a lack of functional inflammasomes increases vulnerability to colitis‐associated colon cancer, enhancing the dual nature of pyroptosis in cancer biology [236, 237, 238] (Figure 3).

Mechanistically, tumor‐suppressive pyroptosis is typically associated with acute, tumor‐cell‐intrinsic gasdermin activation (e.g., GSDME), which promotes immunogenic cell death and enhances antitumor immunity by facilitating immune activation and cytotoxic lymphocyte responses [239]. In contrast, tumor‐promoting effects are more often linked to chronic inflammasome engagement and sustained IL‐1β/IL‐18 signaling, which can maintain a protumor inflammatory milieu, favoring immunosuppressive myeloid recruitment, tissue remodeling, and metastatic dissemination [240, 241].

Unlike apoptosis, which occurs without provoking inflammation, pyroptosis involves the rupture of the plasma membrane and discharge of intracellular contents, initiating a pronounced inflammatory response [242]. Key players, including gasdermins, inflammasomes, and inflammatory cytokines, orchestrate pyroptosis and influence the tumor microenvironment‐immune cell infiltration, epithelial‐to‐mesenchymal transition, and remodeling of the ECM [243]. The impact of pyroptosis on cancer is context‐specific, determined by cell type, genetic factors, and the duration of pyroptotic signaling. Insight into these pathways provides promising strategies for therapeutic interventions aimed at manipulating pyroptosis in cancer treatment. This context dependency mirrors what is described in other sections of this review: while acute pyroptosis contributes to host defense in infections, persistent inflammasome‐driven cytokine release in autoimmune or metabolic disorders can create chronic inflammatory states that may ultimately support tumor initiation or progression.

#### 4.4.1 Breast Cancer

Breast cancer is one of the most common malignancies and exhibits remarkable molecular and clinical diversity [243, 244]. Increasing evidence indicates that pyroptosis‐related molecules play a dual role in this cancer, influencing tumor progression, treatment response, and immune regulation [245]. Elevated expression of GSDMB has been consistently linked to increased metastasis and reduced overall survival, particularly in HER2‐positive tumors, where it also predicts poor responsiveness to targeted therapy [246, 247, 248]. Similarly, GSDMC upregulation correlates with unfavorable prognosis, while activation of Caspase‐8 and PD‐L1 nuclear translocation can induce GSDMC‐dependent pyroptosis after exposure to certain chemotherapeutic drugs such as doxorubicin or actinomycin D [249, 250].

In contrast, GSDME (DFNA5) tends to be silenced by promoter methylation in many breast tumors, especially hormone receptor‐positive subtypes, and its downregulation is associated with lymph node metastasis and chemoresistance [251, 252]. Restoring GSDME expression re‐sensitizes breast cancer cells to paclitaxel and enhances p‐53 mediated pyroptosis, suggesting its potential as both a prognostic and therapeutic biomarker [253, 254]. Members of the p53 family, including p64 and p73, can also transcriptionally activate GSDME, reinforcing its role in tumor suppression [255, 256].

Inflammasome components such as NLRP3, Caspase‐1, and IL‐1β are often elevated in breast cancer tissues, influencing macrophage polarization and immune cell recruitment [257]. Paradoxically, high NLRP3 and IL‐1β expression has been correlated with both increased metastasis and, in some datasets, prolonged survival, depending on molecular subtypes and immune context [258]. Furthermore, P2×7 receptor signaling, through ATP‐mediated activation and ROS generation, promotes pyroptotic and autophagic responses that may enhance immunogenicity and therapeutic sensitivity [258, 259].

Chronic inflammation, obesity, and periodontal infection can further stimulate IL‐1β‐driven chemokines such as CCL2 and CCL5, facilitating macrophage and myeloid‐derived suppressor cell infiltration into tumor tissue [260, 261]. Inhibition of the inflammasome–IL‐1 axis or targeting CCL5 signaling has shown promise in reducing metastasis and restoring immune surveillance [262]. Overall, pyroptosis in breast cancer functions as a double‐edged sword: while excessive inflammatory signaling can support tumor spread, controlled activation of gasdermin and inflammasomes holds potential as an innovative anticancer strategy [263].

#### 4.4.2 Cervical Cancer

Cervical cancer remains one of the most prevalent gynecological malignancies worldwide and continues to rank among the leading causes of cancer‐related mortality in women [264, 265]. Studies using HeLa cells have demonstrated that overexpression of GSDMB induces classical features of pyroptotic cell death [266]. Furthermore, cytotoxic lymphocytes can promote GSDME cleavage through the release of GzmB, thereby triggering pyroptosis in HeLa cells [267]. This observation underscores a reciprocal interaction between GSDME‐mediated pyroptosis and the immune response. In line with this, normal cervical epithelial cells secrete significantly lower amounts of IL‐1β and IL‐18 compared with cervical carcinoma cells, suggesting that pyroptosis‐related cytokines contribute to the proinflammatory microenvironment characteristic of the disease [268].

The sirtuin 1 (SIRT1) deacetylase is markedly upregulated in human papillomavirus (HPV)‐infected cervical cancer cells [269]. Silencing SIRT1 not only enhances the expression of AIM2 and its downstream inflammasome‐associated genes but also induces pyroptotic cell death [270]. These findings position SIRT1 as a potential molecular target for therapeutic intervention in HPV‐associated cervical cancer. Mechanistically, SIRT1 represses the NF‐κB‐dependent transcription of AIM2 by destabilizing RelB mRNA, thereby preventing inflammasome activation [271]. Conversely, SIRT1 knockdown restores RelB stability and facilitates AIM2 inflammasome assembly, culminating in Caspase‐1‐mediated pyroptosis [272]. Given that AIM2 serves as a cytosolic sensor for double‐stranded DNA (dsDNA), including viral DNA from HPV, this pathway may represent a critical axis linking viral persistence, inflammation, and carcinogenesis [272]. Additionally, extracellular vesicles can mediate the intracellular transfer of AIM2 inflammasome components, propagating pyroptotic signaling across tumor cells [273].

Recent evidence has revealed that HPV E7, a viral oncoprotein, suppresses pyroptosis triggered by cytosolic DNA transfection [274]. This occurs through recruitment of the E3 ubiquitin ligase TRIM21, which targets the IFI16 inflammasome for ubiquitination and degradation [274]. The degradation of IFI16 enables HPV to evade innate immune surveillance, providing a mechanism of viral immune escape that contributes to cervical oncogenesis [275]. Targeting this pathway could therefore enhance antiviral immunity and restore inflammasome‐mediated tumor suppression [276].

The NLRP3 inflammasome has also been implicated in the innate immune response of cervical cancer. It is widely expressed in tumor tissues, where its activation can be triggered by ROS, lysosomal disruption, or ion fluxes. In cervical carcinoma cells, ROS act as a primary activator of NLPR3, initiating Caspase‐1‐dependent pyroptosis [277, 278]. Interestingly, AIM2 activation in HPV‐positive cells has been shown to exert tumor‐suppressive effects by promoting pyroptosis, while the same cells release elevated levels of IL‐1β and IL‐18 compared with normal epithelial cells [279]. However, some studies have reported that removal of pyroptosis‐associated cytokines can inhibit tumor growth while simultaneously reducing systemic antitumor immunity [280, 281].

MicroRNA regulation further contributes to pyroptotic control in cervical cancer. Downregulation of miR‐214 in patient tissues correlates with reduced expression of NLRP3 and Caspase‐1 [282]. Conversely, miR‐214 overexpression can reactivate NLRP3 inflammasome signaling and induce pyroptotic death in cervical cancer cells, suggesting a tumor‐suppressive function [283]. Similarly, miR‐145 has been identified as a regulator of GSDMD in the signaling axis, thus representing a potential therapeutic target for restoring pyroptosis and suppressing tumor cell proliferation [284, 285].

Supporting evidence from in vivo and in vitro models has also shown decreased expression of Caspase‐1, GSDMD, and NLRP3 in cervical and endometrial tumor samples compared with normal tissues [286]. Functional restoration of these proteins through gene overexpression or pharmacological induction leads to reduced tumor burden and enhanced pyroptosis [287].

Altogether, these findings indicate that multiple inflammasome pathways, particularly those governed by NLRP3, AIM2, and GSDMD, coordinate the balance between inflammation‐driven tumor promotion and immune‐mediated tumor suppression in cervical cancer. Further elucidation of these networks will be essential for developing pyroptosis‐based therapeutic strategies.

#### 4.4.3 Colorectal Cancer

Colorectal cancer is a multifactorial malignancy influenced by genetic alterations, aging, and lifestyle [288]. Increasing evidence indicates that pyroptosis contributes to both tumor suppression and progression in this cancer, depending on the molecular and microenvironmental context.

Among the gasdermin family, GSDMC promotes tumor proliferation and carcinogenesis, while GSDMD and GSDME show reduced expression in tumor tissue compared with normal colon epithelium [288, 289]. GSDME methylation signatures may serve as diagnostic biomarkers, and lobaplatin can induce ROS/JNK‐mediated pyroptosis via GSDME activation [290]. Conversely, overexpression of lncRNA RP1‐85F18.6 inhibits pyroptosis and supports tumor growth [291].

The inflammasome complex regulates intestinal immune homeostasis and tumor prevention [292]. Loss of inflammasome components in mouse models results in severe inflammation, increased tumorigenesis, and decreased production of IL‐1 and IL‐18. Restoration of NLRP1 expression with DAC suppresses tumor development, enhancing its therapeutic potential [293, 294].

In the AOM/DSS model of colitis‐associated cancer, the absence of NLRP3, ASC, or Caspase‐1 promotes epithelial damage, leukocyte infiltration, and elevated tumor burden due to impaired IL‐18 signaling [295, 296]. Yet, discrepancies among studies suggest that differences in gut microbiota or methodology influence disease outcomes [297, 298]. Overall, active inflammasomes appear to exert protective effects in early inflammation by maintaining epithelial integrity.

IL‐18 plays a dual role, facilitating mucosal repair at early stages but promoting tumor growth when chronically overexpressed [299]. Recombinant IL‐18 alleviates disease severity in Caspase‐1‐deficient mice, supporting its regenerative role [300]. Similarly, AIM2 acts as a DNA sensor that triggers pyroptosis, and its downregulation correlates with poor colorectal cancer prognosis [301, 302].

Recent findings show that reactivation of NLRP3–ASC–Caspase‐1 signaling induces pyroptosis and suppresses cancer progression, while compounds like FL118 enhance these pathways [303]. GSDMD expression is linked to better prognosis and increased chemosensitivity to oxaliplatin (L‐OHP) through LPS‐mediated pyroptosis, suggesting it as a potential predictive biomarker. Likewise. GSDME may serve as a detection marker and therapeutic modulator of pyroptotic death in colorectal cancer [303].

Despite these advances, effective immunotherapies for metastatic colorectal cancer remain limited. Combined strategies involving chemotherapy, surgery, and lifestyle intervention continue to improve survival. Targeting pyroptosis‐related molecules such as GDMs, Caspase‐1, and NLRP3 may open novel therapeutic avenues for colorectal cancer management.

#### 4.4.4 Esophageal Cancer and Gastric Cancer

Chronic reflux and alcohol accumulation exposure promote pyroptosis‐mediated inflammation in esophageal epithelial cells by inhibiting Caspase‐1 and increasing IL‐1β and IL‐18 release [304, 305]. This sustained activation of pyroptotic pathways contributes to esophagitis, which can progress to Barrett's esophagus and eventually esophageal cancer [306, 307]. In Barrett's‐derived cells, LPS triggers TLRN4/NLRP3 inflammasome activation, enhancing mitochondrial ROS, Caspase‐1 cleavage, and pyroptotic cell death [308, 309]. These processes perpetuate mucosal damage and inflammation, aggravating disease progression.

In esophageal squamous cell carcinoma, GSDME expression is markedly elevated and linked to both apoptosis and pyroptosis signaling [288, 310]. However, whether this upregulation drives or merely reflects tumor development remains unclear.

In gastric cancer, multiple gasdermins and inflammasome components exhibit dysregulated expression. GSDMA and GSDMC act as tumor suppressors, while GSDMB tends to promote invasion and proliferation [311, 312, 313]. GSDMD, often reduced in gastric cancer cells, regulates the PI3K/AKT, ERK1/2, and STAT3 pathways; its loss enhances cell‐cycle progression and tumor growth [314, 315, 316]. In contrast, GSDME functions as a p53‐regulated suppressor, and its reactivation can convert apoptosis into pyroptosis, increasing chemosensitivity to agents like 5‐fluorouracil [257].

Inflammasome dysregulation also plays a critical role in gastric tumorigenesis. NLRP3 and NLRC4 are upregulated in gastric cancer and linked to IL‐1β and IL‐18 production [317, 318]. *Helicobacter pylori* infection stimulates these pathways, leading to chronic inflammation, angiogenesis, and epithelial transformation [319, 320]. IL‐1β overexpression enhances tumor growth through recruitment of myeloid‐derived suppressor cells, while IL‐18 promotes vascular remodeling via JNK‐dependent mechanisms [321, 322].

Together, these findings indicate that pyroptosis and inflammasome activation have dual roles in esophageal and gastric cancer, protective in early defense but protumorigenic when chronically stimulated. Understanding the context‐dependent mechanisms may guide new targeted and immunomodulatory therapies.

#### 4.4.5 Glioma

Glioma, particularly glioblastoma (GBM), represents the most aggressive and therapy‐resistant form of primary brain tumors [323]. Despite advances in molecular profiling, prognosis remains poor due to diffuse infiltration, recurrence, and resistance to standard chemotherapy such as temozolomide [324]. One of the key challenges in GBM therapy lies in the tumor's ability to evade apoptosis through DNA repair mechanisms like MGMT activation, promoting treatment resistance [325].

In gliomas, GSDME has been identified as a central effector in pyroptotic signaling, linking Caspase‐3 activation to pore formation and cell lysis [326]. Interestingly, while murine glioma models readily undergo GSDME‐mediated pyroptosis, human GBM cells exhibit partial resistance, potentially due to calcium‐dependent membrane repair mechanisms [326]. This resistance suggests that pyroptosis may not always result in tumor suppression. Indeed, high endogenous expression of GSDME has been associated with enhanced invasion and reduced infiltration of CD8^+^ T cells, indicating a possible protumorigenic role in GBM [326].

Therapeutically, several agents, including arsenic trioxide, benzimidazoles, and melatonin, have shown potential to induce pyroptosis through Caspase‐1, Caspase‐3, or NLRP3 activation [327]. Moreover, microRNAs and nanoparticle‐based systems are emerging as modulators capable of triggering pyroptotic death in glioma cells [328, 329]. Such approaches may sensitize tumors to immunotherapy, as pyroptotic signaling promotes cytokine‐driven immune activation and immune checkpoint engagement.

Overall, pyroptosis represents a double‐edged mechanism in glioma: it can stimulate antitumor immunity, or, conversely, support tumor progression depending on expression context and microenvironmental balance. A deeper understanding of these dynamics could guide the design of novel nanomedicine and combinatorial strategies aimed at selectively harnessing pyroptosis for therapeutic benefit in GBM.

#### 4.4.6 Hepatocellular Carcinoma

Hepatocellular carcinoma, the most common primary liver cancer, remains one of the leading causes of cancer‐related mortality worldwide [330]. Despite improvements in diagnosis and therapy, its progression is often linked to chronic liver injury, cirrhosis, and impaired immune surveillance [331, 332]. The role of pyroptosis in hepatocellular carcinoma has gained increasing attention, although its molecular mechanism remains incompletely understood.

Early studies revealed that Caspase‐1, IL‐1β, and IL‐18 expression levels are markedly reduced in this cancer compared with adjacent normal liver, suggesting that impaired inflammasome activation may contribute to tumor progression [333, 334, 335]. Remarkably, 17β‐estradiol has been shown to activate the NLRP3 inflammasome and induce Caspase‐1‐dependent pyroptosis, thereby exerting antitumor effects in hepatocytes [336, 337].

Similarly, AIM2 inflammasome activation limits tumor growth by inhibiting the mTOR–S6K1 signaling axis, and its downregulation has been associated with poor prognosis and enhanced oncogenic activity [338, 339, 340]. Moreover, GSDME is significantly underexpressed in hepatocellular carcinoma cells, and its upregulation suppresses proliferation, reinforcing its potential as a tumor suppressor gene [341].

Additional studies suggest that autophagy inhibition can enhance pyroptotic death in liver cancer cells, indicating a cross‐regulatory relationship between these two processes [342, 343]. Natural compounds such as berberine have also been shown to induce Caspase‐1‐mediated pyroptosis and reduce hepatocellular carcinoma cell viability [344, 345].

Given the frequent coexistence of hepatic steatosis, fibrosis, and inflammation in this cancer, inflammasome dysregulation may represent a central pathogenic link between chronic liver injury and malignant signaling, particularly through NLRP3, AIM2, or GSDME pathways, which could provide new therapeutic venues for the management of hepatocellular carcinoma.

#### 4.4.7 Lung Cancer

Lung cancer remains the leading cause of cancer‐related mortality worldwide, with non‐small cell lung cancer representing the most prevalent subtype [346]. Recent studies have revealed that GSDMD is upregulated in this cancer, where its elevated expression correlates with greater tumor invasiveness and advanced clinical stages [347, 348]. In contrast, silencing GSDMD suppresses tumor proliferation by attenuating the EGFR/AKT signaling pathway and enhancing apoptotic activity [349]. Interestingly, in the absence of GSDMD, activation of the NLRP3/Caspase‐1 pathway shifts cell death from pyroptosis toward apoptosis, suggesting a molecular pathway interplay between these mechanisms [350].

Furthermore, GSDME appears to determine the mode of Caspase‐3‐induced cell death, where its overexpression promotes pyroptosis, while loss of GSDME contributes to drug resistance and tumor progression. This enhances the importance of gasdermin‐mediated pyroptosis in modulating therapeutic sensitivity [351].

Inflammasome components, including NLRP3, ASC, Caspase‐1, IL‐1β, and IL‐18, are upregulated in aggressive non‐small cell lung cancer cell lines (A549, H1299), and their inhibition facilitates tumor progression [351, 352]. Conversely, activating the ROS/NF‐κB/NLRP3/GSDM axis induces pyroptotic death and limits tumor growth, suggesting its potential as a therapeutic target [353].

Also, lncRNA–XIST has been identified as a key oncogenic regulator that suppresses miR‐355/SOD2/NLRP3‐dependent pyroptosis, thereby promoting the development of this cancer. Restoring this pathway enhances ROS generation and activates inflammasome signaling, reestablishing pyroptotic sensitivity in lung cancer cells [353, 354, 355].

Overall, accumulating evidence indicates that inflammasome‐driven pyroptosis, mediated by gasdermins, caspases, and noncoding RNAs, plays a dual role in lung tumor biology, influencing tumor growth, immune interactions, and response to chemotherapy.

#### 4.4.8 Melanoma

Pyroptosis profoundly shapes melanoma biology and the tumor immune microenvironment [356]. Studies integrating TCGA, GEO, and GTEx datasets have repeatedly shown that expression patterns of pyroptosis‐related genes (such as members of the gasdermin family, inflammasome sensors like AIM2 and NLRP6, and cytokines IL‐1β/IL‐18) stratify patients into high‐ or low‐risk groups with distinct survival outcomes and immune landscapes [357, 358, 359].

Low‐risk tumors typically have an immune‐inflamed phenotype: richer infiltration by CD8^+^ T cells, dendritic cells, and activated macrophages, higher expression of immune checkpoint (PD‐1/PD‐L1, CTLA‐4), and greater predicted responsiveness to immune checkpoint inhibitors (ICIs) [360, 361]. High‐risk tumors tend to be less immune‐infiltrated and show different chemosensitivity profiles as predicted by drug‐response algorithms [362, 363].

Functionally, GSDME has been identified as a tumor suppressor in several melanoma models: its activation (often downstream of Caspase‐3) converts apoptosis into proinflammatory pyroptosis, increasing tumor immunogenicity and enhancing NK and CD8^+^ T‐cell responses [364, 365, 366]. Loss of GSDME can blunt this antitumor immunity, allowing for larger tumor growth [367]. Conversely, chronic or dysregulated inflammasome activity, notably via NLRP3 or NLRP1, can promote recruitment of myeloid suppressor cells and support metastasis in certain contexts, illustrating that inflammasome signals can be either protective or tumor‐promoting depending on timing and cell type [368, 369].

Experimentally, combined targeted therapies (BRAF + MEK inhibitors) have been shown to trigger GSDME‐dependent pyroptosis in melanoma, linking targeted therapy responses to immune activation [370]. Other factors, such as ROS, iron, and noncoding RNAs, modulate pyroptosis sensitivity and may be harnessed to overcome resistance [371]. Altogether, these insights position pyroptosis both as a prognostic biomarker and as a therapeutic lever, one that can be manipulated to convert cold tumors into hot ones but that requires careful tuning to avoid protumor inflammation [372].

#### 4.4.9 Ovarian Cancer

Pyroptosis has recently emerged as an important regulatory mechanism in ovarian cancer, influencing tumor growth, immune activation, and therapeutic response [373]. The long noncoding RNA GAS5 functions as a tumor suppressor by promoting both apoptosis and pyroptosis. Its overexpression activates Caspase‐1 and enhances the secretion of IL‐1β and IL‐18, while silencing GAS5 has the opposite effect, indicating its role in inflammasome activation and immune modulation through disruption of glucocorticoid receptor signaling [374, 375].

Other components of the pyroptotic machinery also contribute to this cancer dynamics. GSDME downregulation has been linked to disease progression, and GSDMD/Caspase‐4 signaling can be pharmacologically reactivated by alpha‐NETA, which triggers pyroptosis and suppresses invasion in epithelial ovarian cancer cells [376, 377]. Conversely, decreased expression of NLRP3, Caspase‐1, and downstream cytokines has been observed in these cancer tissues, suggesting that loss of inflammasome activity supports tumor survival [378, 379].

The lncRNA HOTTIP promotes tumorigenesis by suppressing NLRP1‐mediated pyroptosis and activating the AKT2 pathway, enhancing the complexity of noncoding RNA networks in pyroptotic regulation [291]. Beyond molecular studies, pyroptosis is now recognized as a double‐edged process, capable of inducing antitumor immunity via DAMP release and immune activation, yet potentially tumor‐promoting when inflammation becomes chronic [380].

Overall, integrating pyroptosis into ovarian cancer therapy, through genetic modulation, nanomedicine, or immunotherapy, offers a promising yet intricate frontier. Further studies are needed to balance its antitumor effects with inflammation control and to uncover new molecular targets capable of transforming pyroptosis into a safe and effective therapeutic ally.

#### 4.4.10 Pyroptosis Across Distinct Cancer Types

Pyroptosis, in acute myeloid leukemia, has been linked to inflammasome activation and novel therapeutic targets. The DPP8/9 inhibitor talabostat can trigger CARD8‐dependent pyroptosis in myeloid cells, leading to significant antileukemic effects [381]. Similarly, vitamin B6 has been shown to induce GSDME‐mediated pyroptosis in leukemia models, suggesting that metabolic compounds may be repurposed as immune‐inflammatory modulators [382]. Nonetheless, the inflammatory cytokine surge accompanying pyroptosis, such as IL‐1β and IL‐6 release during CAR T‐cell therapy, raises concerns about cytokine release syndrome, prompting exploration of apoptosis‐pyroptosis balance to optimize efficacy and safety [383, 384, 385].

In cutaneous carcinomas, aberrant activation of inflammasomes such as NLRP3 and AIM2 has been associated with chronic inflammation and keratinocyte transformation [386, 387]. Gasdermin‐mediated membrane permeabilization contributes to immune cell infiltration and cytokine release, which can either promote tumor surveillance or sustain a protumorigenic microenvironment [388, 389]. Dysregulated pyroptotic signaling in keratinocytes may thus act as a critical driver of both tumor initiation and local immune invasion in skin malignancies [390].

Similarly, in intestinal cancers, pyroptosis is closely linked to mucosal inflammation and oxidative stress [294]. Experimental studies show that a deficiency in key inflammasome components, such as Caspase‐1, AIM2, or NLRP1, increases susceptibility to intestinal tumorigenesis by impairing epithelial integrity and immune homeostasis [391, 392]. Conversely, activation of pyroptotic pathways can promote cell death in transformed epithelial cells and enhance antitumor immunity [393]. Gasdermins, particularly GSDMA and GSDMC, display context‐dependent behavior in the intestinal epithelium: while their overexpression favors malignant transformation, their activation induces membrane pore formation and cell lysis, leading to tumor suppression [394, 395].

Furthermore, radiation or genotoxic stress in gastrointestinal tissues may trigger pyroptosis through AIM2 or NLRP3 activation, linking DNA damage to inflammatory cell death [396]. This mechanism not only contributes to epithelial loss but also enhances immune recognition of tumor antigens [396]. Therapeutically, controlled induction of pyroptosis, combined with stander chemoradiotherapy, has shown potential to improve treatment sensitivity in resistant intestinal tumors [397, 398].

Taken together, the evidence across disease categories supports a unifying view: pyroptosis couples stress and danger detection to inflammatory remodeling, but its net impact is determined by magnitude, timing, and tissue context. In cancer, for instance, inflammasome and gasdermin signaling can promote immune surveillance or sustain tumor‐permissive inflammation, while in metabolic‐cardiovascular settings, chronic inflammasome engagement links cellular stress to progressive tissue dysfunction. These dual roles motivate therapeutic strategies that either restrain maladaptive pyroptosis in chronic inflammatory disorders or trigger controlled pyroptosis to enhance antitumor immunity and approach development in the following section.

## 5 Therapeutic Action of Pyroptosis

Therapeutic targeting of pyroptosis requires balancing its inflammatory risk against its immunological and cytotoxic benefits. In this section, it is summarized two complementary intervention logics (i) inhibiting inflammasome–caspase–gasdermin signaling to prevent pathological cytokine release and tissue injury, and (ii) selectively activating pyroptotic execution in cancer or infection o potentiate immune clearance and immunogenic cell death. Also, it further highlights how emerging pharmacological agents and delivery platforms are enabling spatiotemporal control over pyroptosis intensity, improving translational feasibility.

### 5.1 Preclinical Evidence and Clinical Trials Targeting the Pyroptosis Axis

Although pyroptosis is increasingly recognized as a tractable therapeutic axis, direct clinical modulation of the terminal execution step (gasdermin pore formation) remains limited. Most translational and clinical efforts currently target upstream modules that functionally converge on pyroptosis‐associated inflammatory outputs, particularly the NRLP3 inflammasome and downstream IL‐1β/IL‐18 signaling. In parallel, a growing body of preclinical animal work supports the feasibility of targeting gasdermins and inflammasome components to reduce inflammatory tissue injury.

Among upstream interventions, direct NRLP3 inhibitors represent the most pyroptosis‐proximal clinical strategy. For example, dapansutrile (OLT1177) is under active clinical evaluation in inflammatory settings such as acute gout flare (Phase 2/3; NCT05658575) [399] and in Parkinson's disease modification (Phase 2; NCT07157735) [400], where objectives include safety/tolerability and reduction of inflammatory biomarkers in CSF/blood as well as exploratory clinical outcomes. Additional oral NLRP3 inhibitors include inzomelid, evaluated in healthy volunteers and CAPS patients (NCT04015076) [401], and DFV890, assessed in COVID‐19 pneumonia (NCT04382053; completed) [402] and in coronary heart disease with CHIP (NCT06097663; completed) [403]. Notably, published clinical data for DFV890 describe NLRP3 inhibition as a means to reduce IL‐1β/IL‐18 maturation and associated inflammatory cell death outputs.

At the level of downstream cytokine signaling, multiple approved biologics (e.g., IL‐1β neutralization or IL‐1 trapping) provide clinically validated anti‐inflammatory benefit in diseases when inflammasome activation is implicated, even if pyroptosis itself is not directly measured as a primary endpoint. This includes canakinumab in cardiovascular risk reduction (CANTOS; NCT01327846) [404] and IL‐1 pathway blockade in autoinflammatory conditions as recurrent pericarditis (anakinra: NCT02219828; rilonacept RHAPSODY: NCT03737110) [405, 406]. These trials collectively support the clinical importance of the inflammasome–IL‐1 axis that functionally overlaps with pyroptosis‐driven inflammatory amplification.

Finally, gasdermin‐centered targeting is strongly supported preclinically. A *Nature Immunology* study identified disulfiram as an inhibitor of GSDMD pore formation, demonstrating reduced pyroptosis and improved outcomes in inflammatory mouse models [407]. Consistently, animal studies have shown that pharmacological GSDMD inhibition can attenuate inflammatory tissue injury, including vascular pathology models [408]. Together, these translational data indicate that while upstream inflammasome inhibition currently dominates clinical development, future therapies may increasingly incorporate direct modulation of gasdermins or execution‐stage checkpoints to achieve more precise control of pyroptosis. Table 8 summarizes the main action of these preclinical trials.

**TABLE 8:** Representative clinical trials and translational studies targeting the pyroptosis axis (by pathway/mechanism).

Pathway/mechanism (pyroptosis axis)	Agent (target)	Indication	Trial ID (NCT)	Phase/status	Key objective(s)	References
Direct NLRP3 inhibition (upstream inflammasome)	Dapansutrile (OLT1177) (NLRP3 inflammasome inhibitor)	Acute gout flare	NCT05658575	Phase 2/3; recruiting	Efficacy and safety in acute gut flare	[399]
Direct NLRP3 inhibition	Dapansutrile (OLT1177)	Parkinson's disease modification (DAPA‐PD)	NCT07157735	Phase 2; (see record)	Safety/tolerability; effects on inflammation in brain/CSF/blood; exploratory symptom changes	[400]
Direct NLRP3 inhibition	Inzomelid (NLRP3 inhibitor)	Healthy volunteers + CAPS	NCT04015076	Phase 1 (with CAPS cohort); (see record)	Safety/PK/PD; preliminary efficacy in CAPS	[401]
Direct NLRP3 inhibition	DFV890 (NLRP3 inhibitor)	COVID‐19 pneumonia with impaired respiratory function	NCT04382053	Phase 2a: completed	Efficacy and safety vs. standard of care	[402]
Direct NLRP3 inhibition + biomarker strategy	DFV890 (oral) + MAS825 (single s.c.)	Coronary heart disease + CHIP	NCT06097663	Phase 2a: completed	Reduction of inflammatory markers; safety/tolerability	[403]
Downstream IL‐1β blockade	Canakinumab (anti‐IL‐1β)	Secondary prevention CV risk (CANTOS)	NCT01327846	Phase 3: completed (extension noted)	Reduce recurrent of CV events via inflammation targeting	[404]
IL‐1 receptor antagonism	Anakinra (IL‐1R antagonist)	Recurrent idiopathic pericarditis (AIRTRIP)	NCT02219828	Randomized (see record)	Efficacy/safety; recurrent pericarditis	[405]
IL‐1α/ IL‐1β trapping	Rilonacept (IL‐1 trap)	Recurrent pericarditis (RHAPSODY)	NCT03737110	Phase 3; completed	Efficacy/safety; recurrence reduction	[406]
Execution‐stage (gasdermin pore formation)‐preclinical	Disulfiram (GSDMD pore formation inhibitor)	Inflammatory models/septic death in mice	—	Preclinical	Block GSDMD pore formation; reduce pyroptosis	[407]
Execution‐stage (GSDMD inhibition)‐preclinical	Disulfiram	Experimental abdominal aortic aneurysm model	—	Preclinical	Test GSDMD inhibition in vascular inflammatory disease	[408]

### 5.2 Inhibition of Pyroptosis and Inflammasome Activity

Uncontrolled inflammation activation contributes to multiple pathological states, including sepsis, diabetes, neuroinflammation, and viral infections such as HIV [409, 410, 411]. Pyroptosis inhibitors are designed to block these excessive inflammatory responses by preventing caspase activation, gasdermin cleavage, or pore formation.

In HIV infection, Caspase‐1‐mediated pyroptosis drives CD4^+^ T cell depletion and chronic inflammation via IL‐1β release. Inhibiting Caspase‐1 activation effectively prevents HIV‐induced cell death and inflammation [412, 413]. Similarly, sepsis involves systemic pyroptosis triggered by infections; blocking GSDMD or Caspase‐11 in the noncanonical pathway reduces cytokine release and mitigates septic shock and mortality [414, 415]. Diabetes mellitus and its complications are also amplified by chronic NLRP3 inflammasome activation, IL‐1β secretion, and Caspase‐1 signaling, suggesting that pharmacological suppression of these pathways could restore immune homeostasis [416, 417].

Among the studied inhibitors, disulfiram and dimethyl fumarate (DMF) directly interfere with GSDMD activity [418]. Disulfiram, originally used to treat alcoholism, is now recognized as a potent inhibitor of GSDMD‐mediated pyroptosis [419]. It blocks pore formation by covalently modifying Cys191 in human GSDMD, without affecting GSDMD cleavage or upstream caspase activation [420]. Through this mechanism, disulfiram suppresses IL‐1β secretion and protects mice from LPS‐induced sepsis, delaying death even after the inflammatory cascade has begun [420]. The effect is enhanced when combined with Cu (II), improving survival rates [421]. Compared with other cysteine‐reactive inhibitors, such as NSA (a necroptosis inhibitor that blocks GSDMD) and Bay 11–7082 (an NLRP3 inhibitor), disulfiram shows the strongest and most direct action on pore formation rather than caspase inhibition, positioning it as the most effective known GSDMD inhibitor [422, 423, 424].

DMF, another anti‐inflammatory compound, blocks pyroptosis independently of NRD2 or GAPDH [425]. It suppresses LPS‐ and nigericin‐induced LDH and IL‐1β release, reduces GSDMD‐N formation, and prevents membrane pore generation [425]. In vivo, DMF administration protects mice from lethal endotoxin shock by forming 2‐succinyl‐cysteine adducts at Cys191 (human) or Cys192 (mouse) on GSDMD [426]. This modification limits GSDMD interaction with Caspase‐1, reducing pyroptotic death mediated by GSDMD and GSDME. Additionally, DMF decreases CD4^+^, CD8^+^, and myeloid cell infiltration in murine models, showing therapeutic potential in autoimmune and non‐neuroinflammatory diseases, such as multiple sclerosis, FMF, autoimmune encephalitis, and demyelination [427, 428].

Certain natural compounds can also suppress pyroptosis. Scutellarin, a flavone with proapoptotic properties, reduces inflammation by inhibiting NLRP3 activation and modulating PKA signaling [429]. This increases Caspase‐11 phosphorylation, thereby lowering its proteolytic activity toward GSDMD [430]. Blocking PKA or adenylyl cyclase restores pyroptosis, confirming that the PKA–Caspase‐11 axis regulates this inhibitory effect [431]. In addition to scutellarin, several other natural compounds have been reported to modulate inflammasome signaling and pyroptosis. Curcumin and quercetin suppress NLRP3 inflammasome activation by reducing NF‐κB signaling, mitochondrial ROS production, and ASC speck formation, thereby limiting IL‐1β release [432, 433, 434, 435, 436]. Berbamine has also been shown to inhibit inflammasome assembly and downstream Caspase‐1 activation in inflammatory settings [437]. These compounds further support the concept that natural molecules can fine‐tune inflammasome–gasdermin pathways, although scutellarin is enhanced here due to its well‐characterized and direct regulation of the Caspase‐11–GSDMD axis [438].

Emerging biological agents, including anti‐GSDMD nanobodies and antibodies against NINJ1 (a protein involved in terminal membrane rupture), are being developed to prevent uncontrolled DAMP release in acute inflammatory conditions, such as liver injury [439, 440]. Together, these inhibitors offer a broad pharmacological toolbox to fine‐tune pyroptosis and inflammation in clinical settings.

### 5.3 Activation of Pyroptosis in Cancer Therapy

Conversely, activating pyroptosis in cancer cells can induce potent immunogenic cell death, releasing cytokines and DAMPS (IL‐1β, IL‐18, HMGB1) that enhance immune surveillance and improve responses to therapy [441]. Many chemotherapeutic drugs, including doxorubicin, cisplatin, actinomycin D, mitoxantrone, and etoposide, can trigger Caspase‐3‐mediated cleavage of GSDME, transforming apoptosis into pyroptosis [442]. In tumors where GSDME is silenced by promoter methylation, agents like decitabine restore its expression and sensitize cells to pyroptotic death [443].

Targeted inhibitors such as BRAF or MEK blockers in melanoma and dasatinib in lung cancer also initiate pyroptosis by engaging mitochondrial and caspase cascades [444, 445]. The subsequent release of HMBG1 and other alarmins stimulates macrophage infiltration and cytotoxic T‐cell activation, reshaping the tumor microenvironment toward an inflamed, antitumor phenotype [446, 447].

Immunotherapy further amplifies these effects. ICIs, including anti‐PD‐1 or CTLA‐4 antibodies, may enhance pyroptotic signaling and immune infiltration. Inhibiting enzymes such as DPP8/9 or USP48 augments this synergy, providing opportunities for combined strategies where pyroptosis acts as a catalyst for immune activation [448, 449, 450]. Furthermore, microbial components such as LPs or peptidoglycans can stimulate inflammasome activity in tumor cells, linking infection‐associated inflammation with antitumor immunity [451].

### 5.4 Nanotechnology and Pyroptosis‐Based Therapies

Nanomedicine offers a platform to selectively control pyroptosis while minimizing systemic toxicity [452]. Small‐molecule nanocarriers containing chemotherapeutics or photosensitizers can activate the Caspase‐3–GSDME pathway selectively in tumor tissues, promoting immune activation with reduced side effects [453, 454]. Sequential strategies, such as decitabine pretreatment followed by cisplatin‐loaded liposomes, successfully reinduce GSDME expression and pyroptosis, accompanied by IL‐1β and IL‐18 secretion [455, 456].

Polymer‐based nanocarriers, liposomes, and photoresponsive nanoparticles have also demonstrated synergistic effects by triggering pyroptosis while stimulating dendritic cells and cytotoxic lymphocytes [457, 458]. Nucleic acid nanomedicines provide additional precision: mRNA or siRNA nanoparticles targeting inflammasome components or encoding GSDMB‐N domains can directly induce pyroptosis in tumor cells [459]. When combined with ICIs or RIG‐1 agonists, these nanomedicines amplify immune responses and suppress tumor growth in preclinical models [460, 461].

Table 9 summarizes the main actions of the therapies in pyroptosis. Finally, these therapeutic advances reinforce the central message of this review: pyroptosis is most clinically actionable when its upstream regulators and gasdermin‐dependent outcomes are interpreted in a context‐specific manner, providing a rationale for the integrative conclusions outlined below.

**TABLE 9:** Main pharmacological modulators of pyroptosis and their therapeutic potential.

Compound/strategy	Primary target	Mechanism of action	Therapeutic context/effects	References
Disulfiram	GSDMD	Covalently modifies cysteine residues to block pore formation without affecting GSDMD cleavage; inhibits IL‐1β release and caspase activity indirectly	Protects against LPS‐induced sepsis; potential therapy for cancer and inflammatory disorders; synergistic effect when combined with Cu (II)	[419]
Dimethyl fumarate (DMF)	GSDMD and GSDME	Induces succination at Cys191/192, inhibiting GSDMD binding to Caspase‐1 and pore formation; reduces LDH and IL‐1β release	Approved for multiple sclerosis; effective in experimental autoimmune encephalitis, familial Mediterranean fever, and inflammatory conditions	[425]
Necrosulfonamide (NSA)	GSDMD	Cysteine‐reactive inhibitor that blocks GSDMD‐mediated pyroptosis and necroptosis	Reduces inflammation and cell lysis in inflammasome‐associated diseases	[422]
Bay 11–7082	NRLP3 inflammasome	Inhibits inflammasome assembly and Caspase‐1 activation	Attenuates pyroptosis in sepsis and inflammatory disorders	[423]
MCC950	NLRP3 inflammasome	Prevents ATP‐dependent NLRP3 activation and IL‐1β	Reduces systemic inflammation and tissue injury in inflammasomopathies	[424]
Scutellarin	Caspase‐11 via PKA pathway	Enhances Caspase‐11 phosphorylation through PKA activation, reducing GSDMD cleavage	Decreases macrophage pyroptosis; potential use in inflammatory and cardiovascular diseases	[429]
Anti‐GSDMD nanobodies	GSDMD	Ind to GSDMD pores to prevent further oligomerization and cytokine release	Experimental therapy to control excessive inflammation and pyroptosis in vivo	[439, 440]
Anti‐NINJ1 antibodies	NINJ1 protein	Prevent NINJ1 oligomerization, blocking DAM release (IL‐18, HMGB1, mtDNA)	Protects against acute liver injury and systemic inflammation in murine models	[439, 440]
Chemotherapeutics (doxorubicin, cisplatin, actinomycin D)	Caspase‐3 → GSDME axis	Induces Caspase‐3‐ediated cleavage of GSDME, converting apoptosis to pyroptosis	Promote immunogenic cell death and enhance immune response in cancer therapy	[442]
DNA methylation inhibitors (decitabine)	GSDME promoter region	Reactivates silenced GSDME to restore pyroptosis sensitivity	Sensitizes tumor cells to chemotherapy‐induced pyroptosis	[444, 445]
Nanomedicine strategies (liposomes, polymeric and photoresponsive nanoparticles)	Caspase‐gasdermin signaling in tumor cells	Targeted delivery of pyroptosis‐inducing drugs; control of spatiotemporal activation	Boosts tumor immunity, enhances ICI response, minimizes systemic toxicity	[455, 456]

## 6 Conclusions

Pyroptosis has emerged as a central and highly dynamic form of regulated cell death that uniquely links cellular demise to inflammatory signaling. Throughout this review, it is enhanced how diverse molecular pathways, centered on the inflammasome, inflammatory caspases, and the gasdermin family proteins, converge to execute pyroptosis through multiple activation routes. This mechanistic versatility distinguishes pyroptosis from other PCD modalities and underlies its prominent role in shaping innate immune responses, tissue homeostasis, and inflammatory pathology.

A key concept emphasized in this review is that pyroptosis does not function in isolation but is deeply embedded within a broader network of regulated cell death programs. Extensive crosstalk with apoptosis, necroptosis, ferroptosis, NETosis, and the integrative process of PANoptosis allows shared molecular regulators, such as caspases, inflammasomes, and gasdermins, to generate context‐dependent outcomes. This interconnectedness provides a mechanistic explanation for the dual nature of pyroptosis, which can be protective during acute infection or immune surveillance, yet detrimental when aberrantly or chronically activated, contributing to autoimmune, metabolic, cardiovascular, and neurodegenerative diseases.

From a translational perspective, pyroptosis represents a dual‐edged therapeutic target. Current strategies largely focus on upstream modulation of inflammasomes or downstream cytokine signaling to mitigate excessive inflammation, while emerging approaches aim to harness controlled pyroptotic cell death to enhance immunogenic responses, particularly in cancer. However, the clinical exploitation of pyroptosis requires precise spatiotemporal regulation, given the narrow boundary between beneficial immune activation and pathological tissue damage. These challenges enhance the need for improved biomarkers, cell‐type‐specific targeting strategies, and a deeper understanding of pyroptosis execution dynamics. Key challenges in the field include the lack of specific biomarkers to distinguish pyroptosis from other inflammatory cell death programs, limited tools to selectively modulate gasdermin activity in vivo, and the difficulty of translating preclinical findings into safe clinical interventions.

Looking ahead, several critical questions remain unresolved. Future research should focus on delineating the temporal regulation of pyroptosis, including sublytic versus lytic outcomes, defining cell‐ and tissue‐specific gasdermin functions, and integrating pyroptosis into systems‐level models of inflammatory and cell death signaling networks. Advances in these areas will be essential for translating mechanistic insights into safe and effective therapeutic interventions. Collectively, continued interdisciplinary investigation will further establish pyroptosis as a pivotal biological process and a promising target in the treatment of human disease.

## Author Contributions

DLB, PDCM, CGM, OFM, MNMA, SBB, LLG, CTF, RAH, TRG, ILU, MDA, AGC, MAS, DCP, JVS, MAM, RDP, and MAO conceived the idea and structure of the review. DLB, PDCM, CGM, OFM, MNMA, SBB, LLG, CTF, RAH, TRG, ILU, MDA, AGC, MAS, DCP, JVS, MAM, RDP, and MAO drafted the manuscript and integrated all sections. All authors critically revised the manuscript for intellectual content and approved the final version.

## Funding

The study was supported by the Comunidad de Madrid (P2022/BMD‐7321), ProACapital, and HALE KULANI, S. L. and MJR.

## Ethics Statement

The authors have nothing to report.

## Conflicts of Interest

The authors declare no conflicts of interest.

## Data Availability

Data sharing is not applicable to this article as no new data were generated or analyzed in this study.
